# The overexpression of OsACBP5 protects transgenic rice against necrotrophic, hemibiotrophic and biotrophic pathogens

**DOI:** 10.1038/s41598-020-71851-9

**Published:** 2020-09-10

**Authors:** Saritha Panthapulakkal Narayanan, Shiu-Cheung Lung, Pan Liao, Clive Lo, Mee-Len Chye

**Affiliations:** School of Biological Sciences, The University of Hong Kong, Pokfulam Road, Pokfulam, Hong Kong China

**Keywords:** Plant sciences, Plant stress responses, Biotic

## Abstract

The most devastating diseases in rice (*Oryza sativa*) are sheath blight caused by the fungal necrotroph *Rhizoctonia solani,* rice blast by hemibiotrophic fungus *Magnaporthe oryzae*, and leaf blight by bacterial biotroph *Xanthomonas oryzae* (*Xoo*). It has been reported that the Class III acyl-CoA-binding proteins (ACBPs) such as those from dicots (Arabidopsis and grapevine) play a role in defence against biotrophic pathogens. Of the six Arabidopsis (*Arabidopsis thaliana*) ACBPs, AtACBP3 conferred protection in transgenic Arabidopsis against *Pseudomonas syringae,* but not the necrotrophic fungus, *Botrytis cinerea.* Similar to Arabidopsis, rice possesses six ACBPs, designated OsACBPs. The aims of this study were to test whether OsACBP5, the homologue of AtACBP3, can confer resistance against representative necrotrophic, hemibiotrophic and biotrophic phytopathogens and to understand the mechanisms in protection. Herein, when OsACBP5 was overexpressed in rice, the OsACBP5-overexpressing (OsACBP5-OE) lines exhibited enhanced disease resistance against representative necrotrophic (*R. solani* & *Cercospora oryzae*)*,* hemibiotrophic (*M. oryzae* & *Fusarium graminearum*) and biotrophic (*Xoo)* phytopathogens*.* Progeny from a cross between OsACBP5-OE9 and the jasmonate (JA)-signalling deficient mutant were more susceptible than the wild type to infection by the necrotroph *R. solani.* In contrast, progeny from a cross between OsACBP5-OE9 and the salicylic acid (SA)-signalling deficient mutant was more susceptible to infection by the hemibiotroph *M. oryzae* and biotroph *Xoo*. Hence, enhanced resistance of OsACBP5-OEs against representative necrotrophs appears to be JA-dependent whilst that to (hemi)biotrophs is SA-mediated.

## Introduction

Despite the existence of natural defence mechanisms in plants, crops can become susceptible to bacterial and fungal pathogens which cause negative impacts on food security and food safety worldwide^1^. This happens when the virulence factors of a pathogen overwhelm the defence responses of a specific host plant^2^. Phytopathogens are classified into necrotrophs, hemibiotrophs and biotrophs based on their interaction with the host. Necrotrophic pathogens rapidly kill host tissues by secreting toxins and survive on the dead remains, whereas biotrophs derive nutrients from living cells and therefore sustain host viability^3,4^. Hemibiotrophic pathogens exhibit both forms of nutrient acquisition, starting from the suppression of host immune system in a biotrophic phase, followed by a later necrotrophic phase during which the pathogen induces host cell death by its toxin production^5^. Thus, necrotrophic pathogens tend to exert a more damaging effect on host plants than biotrophic pathogens and are deemed more detrimental to crop productivity^4^.

Necrotrophic soil-borne phytopathogenic fungi such as *Fusarium* and *Rhizoctonia* infect prominent economically important monocot crops such as rice (*Oryza sativa*), wheat and maize^1,6,7^. *Fusarium solani* infection resulted in a 10–30% drop in yield for pulse crops following severe root-rot^8^. In Japan, soybean foliar blight arising from the necrotroph *Rhizoctonia solani* AG-1 culminated to around 70% decrease in yield^9^. It has been reported that necrotrophic pathogens have a higher economic impact on agriculture over biotrophic pathogens^10^. Reduction in Australian wheat and barley production from necrotrophic pathogen diseases such as tan spot, and *Stagonospora nodorum* blotch, respectively, significantly exceeded that from wheat rusts and mildews caused by biotrophic pathogens^10^. In addition, the necrotroph, *Botrytis cinerea,* infects nearly all crop plants and creates global annual economic loss from $10 to $100 billion^11^. To safeguard a stable food supply to an escalating global population, there is an urgent need to control fungal diseases. To this end, proteins that can defend plants from plant pathogens need to be identified and tested for efficacy in transgenic monocots.

The acyl-CoA-binding proteins (ACBPs) represent a major group of proteins associated with acyl-CoA ester transfer in eukaryotes^12^. The *Arabidopsis thaliana* ACBPs bind to long-chain acyl-CoA esters and function in plant growth, development and stress responses^13–28^. Even though the expression of *AtACBP3* was upregulated by both bacterial biotroph, *Pseudomonas syringae* pv *tomato* DC3000, and fungal necrotroph, *B. cinerea*, transgenic Arabidopsis AtACBP3-overexpressing (OE) lines were conferred protection only against *P. syringae*^17^. Moreover, *atacbp3* was susceptible to *P. syringae,* hemibiotrophic fungal pathogen *Colletotrichum higginsianum* and the fungal necrotroph *B. cinerea* in comparison to the wild type (WT)^29^. It appears that both the overexpression and loss of AtACBP3 affected basal defence against the fungal necrotroph *B. cinerea*^17,29^, suggesting that susceptibility of mutant lines to a pathogen does not always correspond to resistance in OE lines.

A homologous Class III ACBP from *Vitis vinifera* (grape) had displayed enhanced resistance to *P. syringae* when overexpressed in Arabidopsis^30^. The same study revealed that transgenic Arabidopsis VvACBP-OEs were more tolerant against hemibiotrophic fungal pathogen *C. higginsianum* infection in detached leaves, but the response of Arabidopsis VvACBP-OEs against necrotrophic pathogens was not reported^30^. When compared to ACBPs from dicotyledonous plants^17,29,30^, ACBPs from monocotyledonous plants such as rice are less well understood. Rice ACBP5 (OsACBP5) resembles AtACBP3 in being classified as the sole rice Class III ACBP, with the acyl-CoA-binding (ACB) domain located at the *C*-terminus unlike the other three classes^31^. Previous studies revealed that the ACB domain of OsACBP5 binds to 18:3-acyl-CoA ester^31^, and interestingly the 18:3 fatty acid (FA) is associated with resistance to the biotroph *P. syringae* in tomato by activating NADPH oxidase in reactive oxygen species (ROS) production^32^. The rise in ROS initiates the biosynthesis of the plant hormone salicylic acid (SA) leading to hypersensitive response against the pathogen^32,33^.

Phytohormones such as SA and jasmonic acid (JA) are well known in regulating plant defence^34^. SA is synthesized from chorismate, a primary metabolite, through two enzymatic pathways, one involving PHENYLALANINE AMMONIA LYASE and the other ISOCHORISMATE SYNTHASE^35–38^. Pattern- and effector-triggered immunity activates SA biosynthesis upon recognition of pathogen-associated molecular patterns (PAMPs) and effectors of pathogens, respectively^36,39^. The regulatory protein NONEXPRESSOR OF PATHOGENESIS-RELATED GENES1 (NPR1) mostly controls the SA-signalling pathway^36^. NPR1, triggered by SA, functions as a transcriptional coactivator of the *PATHOGENESIS-RELATED* (*PR*) genes^36,40,41^. PR proteins demonstrate antimicrobial activity and *PR-1* represents an important marker for SA-responsive gene expression^36,42^, while the activation of *PR* genes leads to SA-mediated defence^36^.

JA and its structurally-related compounds are synthesized by the oxylipin biosynthesis pathway following pathogen invasion^43^. JA carboxyl methyl transferase catalyses the methylation of JA to methyl jasmonate (MeJA)^44^. JA-amidosynthetase (JAR1) catalyses the production of JASMONYL-ISOLEUCINE (JA-Ile) by conjugation of JA to the amino acid isoleucine^45^. CORONATINE INSENSITIVE1 (COI1), JAR1 and JASMONATE INSENSITIVE1/MYC2 (JIN1/MYC2) are the key components involved in JA-dependent defence responses in plants. COI1 interacts with SKP1/Cullin counterparts and forms the SKP1–Cullin–F-box (SCF)^COI1^ubiquitin E3 ligase complex. The SCF complex mediates protein degradation which is essential for JA-dependent defence responses^46^. The expression of JA-related genes is transcriptionally regulated by the JIN1/MYC2 transcription factor^47^. Jasmonate zim-domain (JAZ) repressor proteins interact with the JIN1/MYC2 transcription factor to suppress the expression of JA-associated genes^47^. COI1 interacts with JAZ proteins to form the COI1-JAZ complex^48^. COI1-JAZ functions as a JA-Ile receptor in the SCF complex^49^. Degradation of the JAZ repressor protein is mediated by the interaction of JA-Ile with COI1^50^. JAZ protein degradation results in the stimulation of JA-related genes by JIN1/MYC2 transcription factors leading to plant defence^50^.

While the JA pathway generally provides protection against necrotrophic pathogens, the SA pathway is normally associated with resistance to biotrophic pathogens^47,51,52^. Several studies have demonstrated that the SA pathway functions antagonistically to the JA-signalling pathway^51,53^. Hence, enhanced tolerance to biotrophic infection is often associated with elevated susceptibility to necrotrophs and vice versa^52,54^ as demonstrated in AtACBP3-OEs^17^. The cross talk between SA and JA has been clarified in dicots such as Arabidopsis whereby suppression of the JA pathway by SA functions downstream of the E3 ubiquitin-ligase SCF complex, which targets JAZs for proteasome-mediated degradation^55^. In comparison, the antagonistic interaction between the SA- and JA-signalling pathways is less well understood in the monocots^56,57^. Therefore, it would be pertinent to decipher the role of OsACBP5 in plant defence, given its ability to bind 18:3-acyl-CoA ester possibly associated with phytohormone signalling.

Meng et al*.* had earlier shown in quantitative real-time PCR (qRT-PCR) analysis that of the six *OsACBPs*, only *OsACBP5* mRNA expression was upregulated following infection with the hemibiotrophic rice blast fungal pathogen, *Magnaporthe oryzae*^31^. Rice blast is a destructive fungal disease causing a 30% decline in rice production globally^58^. Our recent study demonstrated that overexpression of OsACBP5 in transgenic Arabidopsis conferred resistance to representative necrotrophic (*R. solani, B. cinerea, Alternaria brassicicola*), hemibiotrophic (*Colletotrichum siamense*) and biotrophic (*P. syringae*) phytopathogens through cell wall-mediated defence as well as SA- and JA-mediated defence pathways^59,60^. Given the need for crops to be protected against necrotrophic fungal pathogens, the present study follows up on our initial investigations on *OsACBP5*^31^. To this end, transgenic rice lines overexpressing OsACBP5 were generated and tested against representatives derived from three groups of phytopathogens (the necrotrophs, hemibiotrophs and biotrophs). Transgenic rice harbouring *OsACBP5pro::GUS* were also produced to examine *OsACBP5* regulation.

## Results

### Transgenic rice OsACBP5-OEs showed enhanced tolerance against two necrotrophic fungal pathogens, *R. solani* and *C. oryzae*

**Figure 1:**
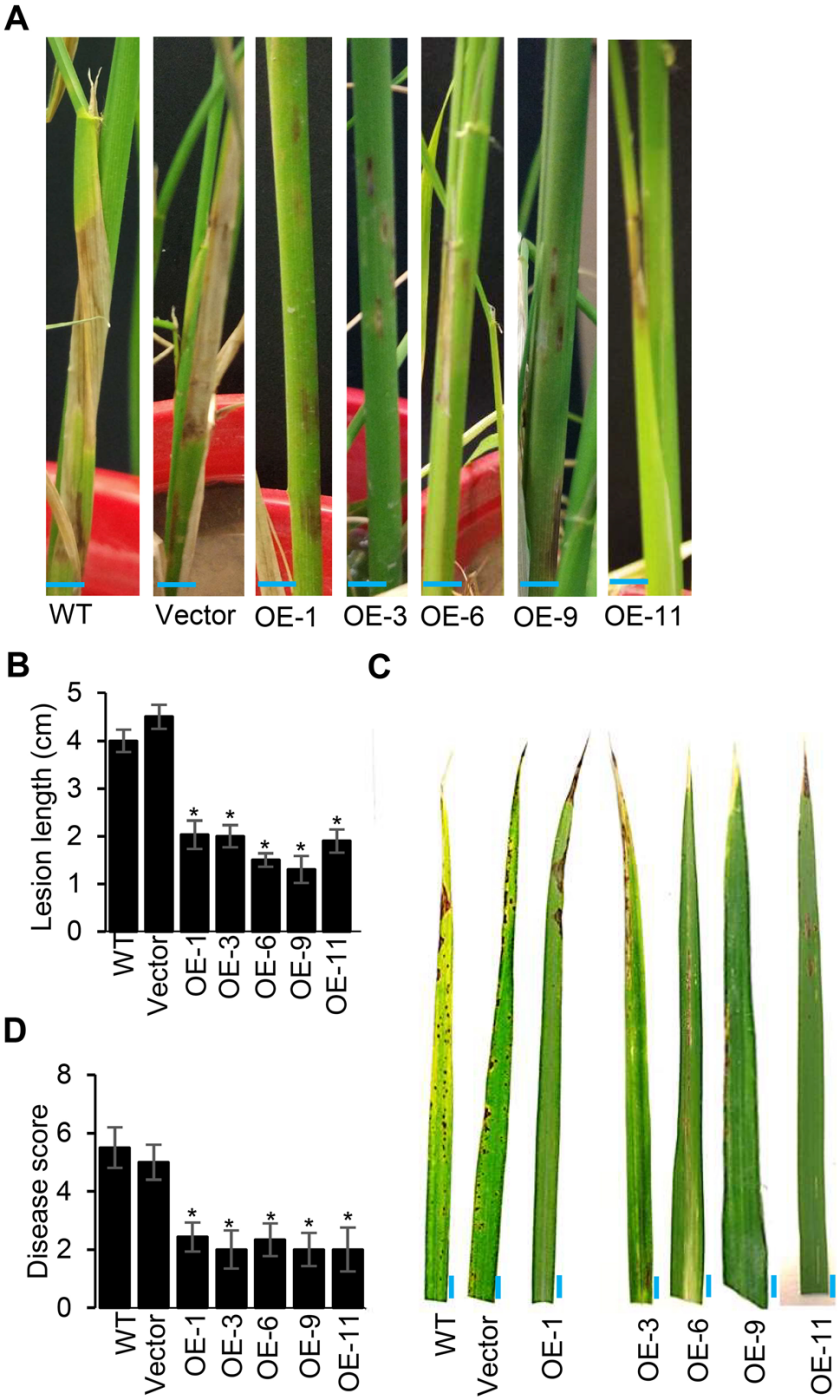
OsACBP5-OE transgenic rice plants displayed improved resistance against fungal necrotrophs*.* (**A**) Sheath blight symptoms on five-week-old WT, vector-transformed control and OsACBP5-OE (OE-1, OE-3, OE-6, OE-9, OE-11) rice inoculated with *R. solani*. Blue bars = 1 cm. (**B**) Lesion length following *R. solani* infection in WT, vector-transformed control and OsACBP5-OEs 14 days-post-inoculation (dpi). (**C**) Disease phenotype following *C. oryzae* infection on three-week-old WT, vector-transformed control and OsACBP5-OEs at 9 dpi. Blue bars = 7 mm. (**D**) Disease scores from *C. oryzae-*infected leaves. Data points represent means ± SD from three independent experiments. Asterisks indicate significant difference (*P* < 0.05) in comparison to the controls by Student’s *t-*test.

Transgenic rice lines overexpressing OsACBP5 (OsACBP5-OEs) were generated and verified by western blot analysis (Supplemental Fig. S1). To explore the function of OsACBP5 in defence, the resistance level of transgenic rice OsACBP5-OEs (OE-1, OE-3, OE-6, OE-9 and OE-11) was first evaluated against sheath blight disease, a severe fungal disease in rice caused by *R. solani,* using sheath inoculation assays. While typical disease lesions on the WT and vector (pCAMBIA1304)-transformed plants were observed, fewer disease lesions appeared on transgenic rice OsACBP5-OEs (Fig. 1A). At 14 days-post-inoculation (DPI), the lesion lengths on the sheaths of WT and vector-transformed plants were 4 cm and 4.5 cm, respectively, while the average lesion length on sheaths of the OsACBP5-OEs was 1.6 cm, representing an approximately twofold reduction in lesion length in comparison to the controls (Fig. 1B). Consistent results were observed when transgenic rice OsACBP5-OEs were infected with the necrotroph *C. oryzae* causing narrow brown leaf spot in rice plants (Fig. 1C). The average disease scores in WT and vector-transformed plants at 9 DPI were 5.5 and 5, respectively, while the average disease score on OsACBP5-OEs was 2.3, resulting in an approximately 2.5-fold reduction in comparison to the controls (Fig. 1D). Taken together, these results suggest that the overexpression of OsACBP5 in transgenic rice conferred enhanced resistance to necrotrophs, *R. solani* and *C. oryzae,* in comparison to WT and vector-transformed plants.

### Transgenic rice OsACBP5-OEs were more resistant to two hemibiotrophic fungal pathogens, *M. oryzae* and *Fusarium graminearum*

**Figure 2:**
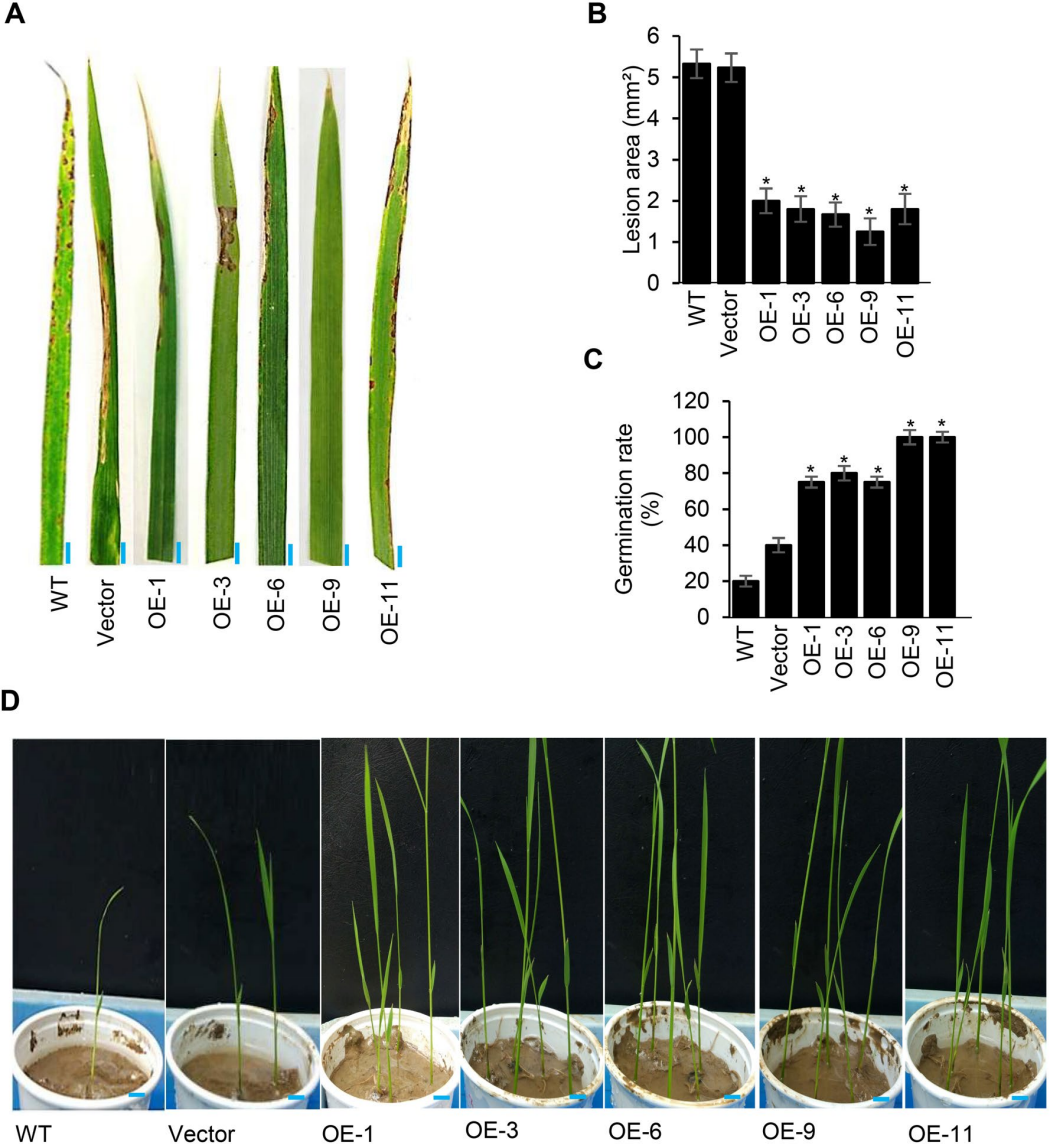
Increased resistance of OsACBP5-OE transgenic rice plants to hemibiotrophic fungal infection. (**A**) Rice blast symptoms after inoculation with *M. oryzae* on 3-week-old WT*,* vector-transformed control and OsACBP5-OE (OE-1, OE-3, OE-6, OE-9, OE-11) at 7 days-post-inoculation (dpi). Blue bars = 5 mm. (**B**) Lesion area on WT, vector-transformed control and OsACBP5-OEs inoculated with *M. oryzae*. (**C**) Germination rate of WT*,* vector-transformed control and OsACBP5-OE rice plants grown from the seeds inoculated with *F. graminearum* at 7 dpi. (**D**) WT*,* vector-transformed control, and OsACBP5-OE rice plants germinated from *F. graminearum-*infected seeds. Blue bars = 1 cm. Data points represent means ± SD from three independent experiments. Asterisks indicate significant difference (*P* < 0.05) in comparison to the controls by Student’s *t-*test.

The resistance level of transgenic rice OsACBP5-OEs was next investigated against blast disease caused by the hemibiotroph *M. oryzae.* When spray inoculation (Fig. 2A) was performed to explore the resistance of OsACBP5-OEs against *M. oryzae*, the average lesion areas in WT and vector-transformed plants at 7 dpi were 5.3 mm^2^ and 5.2 mm^2^, respectively, while the average lesion area on OsACBP5-OEs was 1.7 mm^2^, resulting in approximately 50% reduction in lesion area (Fig. 2B). When the seeds of WT, vector-transformed control and OsACBP5-OEs were imbibed with conidial suspensions of seed-infecting hemibiotroph, *F. graminearum,* the OsACBP5-OEs exhibited 80–100% of seed germination rates, whereas those of WT and vector-transformed controls were 20% and 40%, respectively, suggesting that OsACBP5-OEs were more resistant to *F. graminearum* infection (Fig. 2C,D).

### Transgenic rice OsACBP5-OEs conferred protection against biotrophic bacterial pathogen *Xanthomonas oryzae*

**Figure 3:**
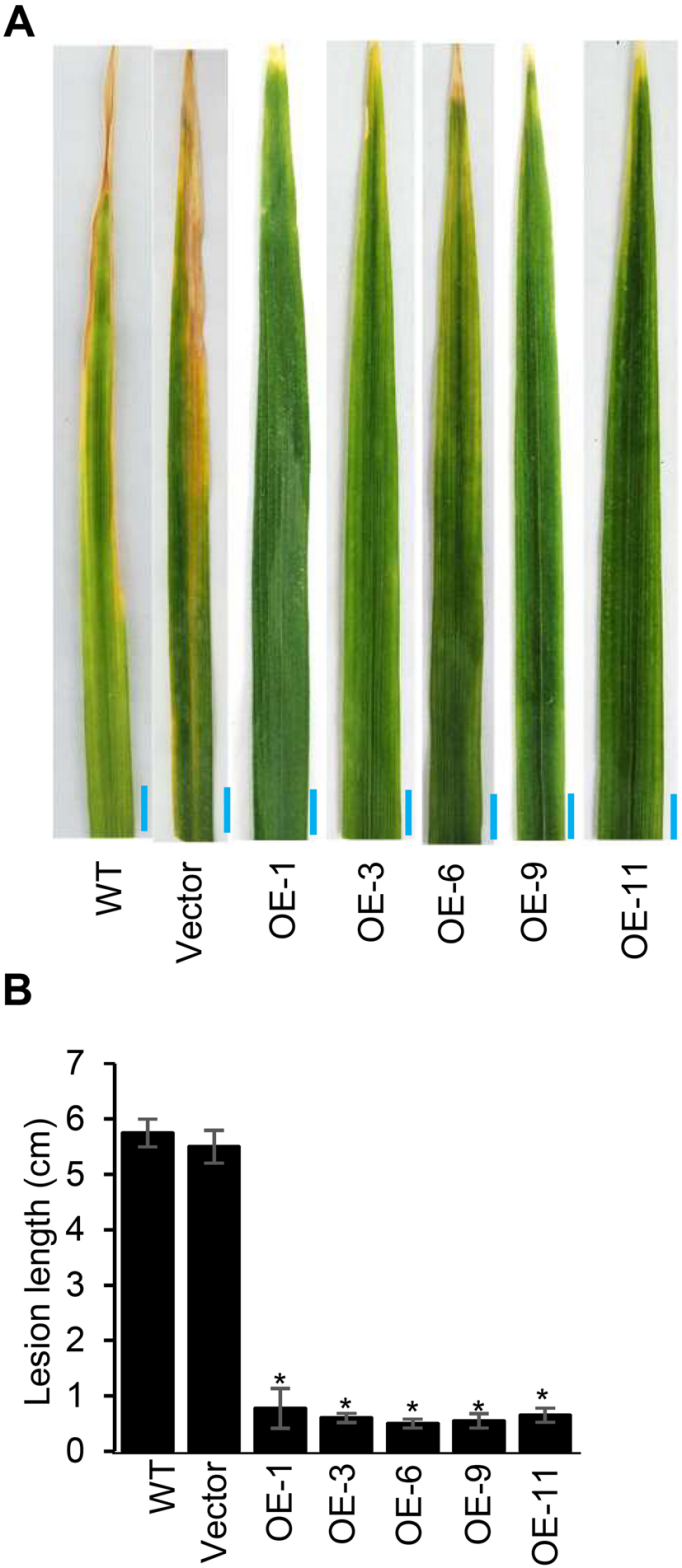
OsACBP5-OE transgenic rice plants are more resistant to the biotrophic bacterial pathogen *Xoo* infection. (**A**) Disease symptoms on three-week-old WT, vector-transformed control and OsACBP5-OE (OE-1, OE-3, OE-6, OE-9, OE-11) plants inoculated with *Xoo*. Leaves were photographed 14 days-post-inoculation. Blue bars = 5 mm. (**B**) Lesion length after inoculation with *Xoo* in WT, vector-transformed control and OsACBP5-OEs*.* Data points represent means ± SD from three independent experiments. Asterisks indicate significant difference (*P* < 0.05) in comparison to the controls by Student’s *t-*test.

When transgenic rice OsACBP5-OEs were evaluated against the bacterial leaf blight disease caused by *Xanthomonas oryzae* pv. *oryzae* (*Xoo*) using the leaf-clipping method (Fig. 3A), the average lesion length on OsACBP5-OEs at 14 dpi was 0.7 cm, while the average lesion lengths in WT and vector-transformed plants were 5.7 cm and 5.5 cm, respectively, representing a fivefold reduction in disease development (Fig. 3B). These results indicate that OsACBP5-OEs displayed enhanced tolerance to *Xoo*.

### SA and JA levels in transgenic rice OsACBP5-OEs were elevated

**Figure 4:**
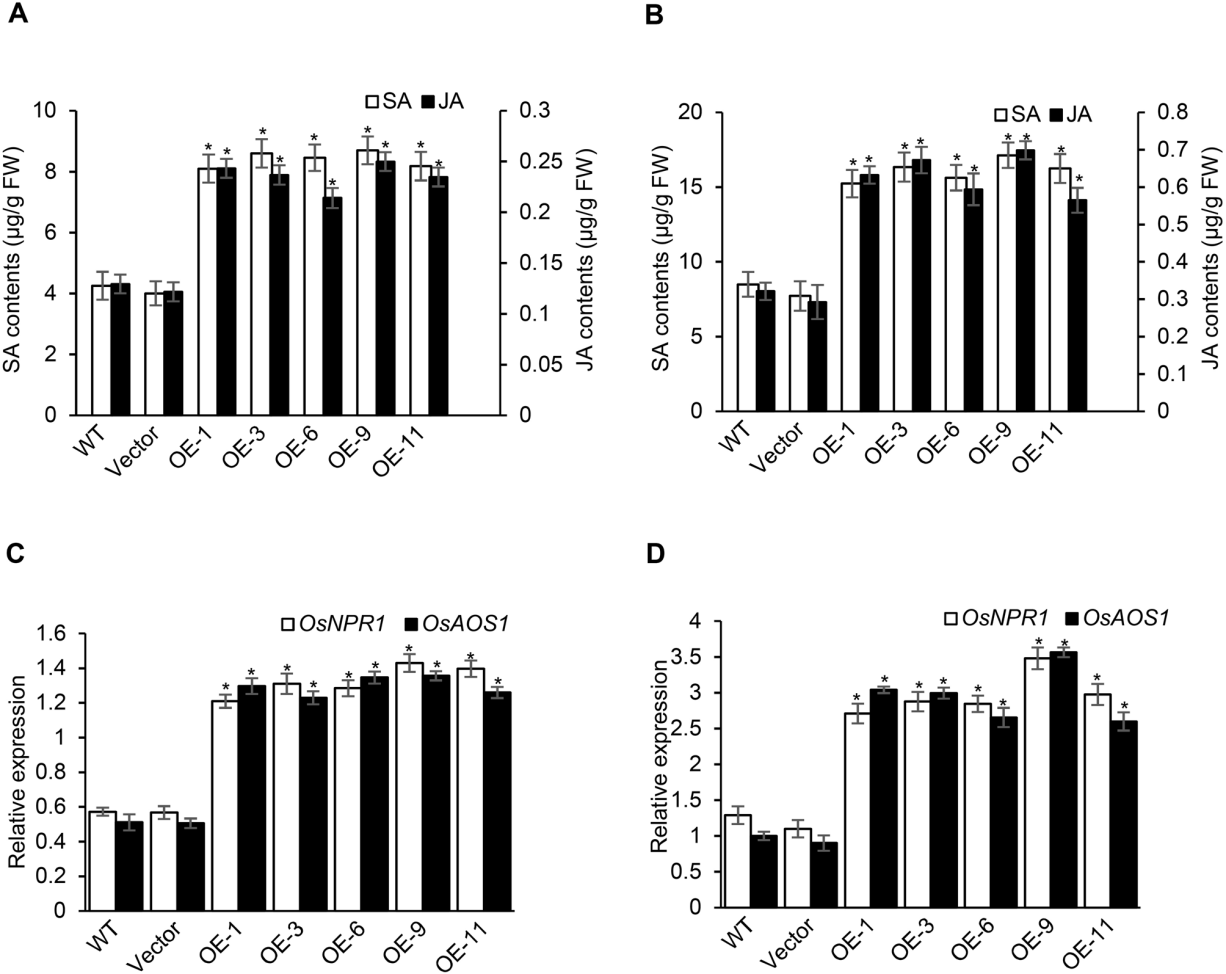
OsACBP5-OE transgenic rice plants displayed elevated SA and JA level. Total SA and JA were extracted from uninfected (**A**) and *R. solani-*infected (**B**) WT, vector-transformed control and OsACBP5-OEs (OE-1, OE-3, OE-6, OE-9, OE-11) and analyzed by GC-MS. qRT-PCR analyses showing the expression of *OsNPR1* and *OsAOS1* in uninfected (**C**) and *R. solani-*infected (**D**) WT, vector-transformed control and OsACBP5-OEs. Data points represent means ± SD from three independent experiments. FW, fresh weight. Asterisks indicate significant difference (*P* < 0.05) in comparison to the controls by Student’s *t-*test.

As SA and JA play important roles in regulating immune responses in rice^61^, possible relationship between SA and JA in enhanced pathogen-resistance in OsACBP5-OEs, was investigated by SA and JA content measurements using gas chromatography-mass spectrometry (GC-MS) on uninfected and *R. solani*-infected plant samples. A two-fold increase in endogenous SA was shown in OsACBP5-OEs in comparison to the controls (Fig. 4A,B). Similarly, the endogenous JA content in OsACBP5-OEs were two-fold higher than the controls (Fig. 4A, B). When qRT-PCR was performed, the expression of *OsNPR1,* an SA*-*signalling regulatory gene, and *ALLENE OXIDE SYNTHASE1* (*OsAOS1*) encoding allene oxide synthase in JA biosynthesis, were upregulated in uninfected and *R. solani*-infected plant samples of OsACBP5-OEs in comparison to that of controls (Fig. 4C,D). These results indicate that both SA- and JA-signalling pathways are activated in OsACBP5-OEs.

### Protection in rice OsACBP5-OE*s* are dependent on both SA- and JA-signalling pathways

**Figure 5:**
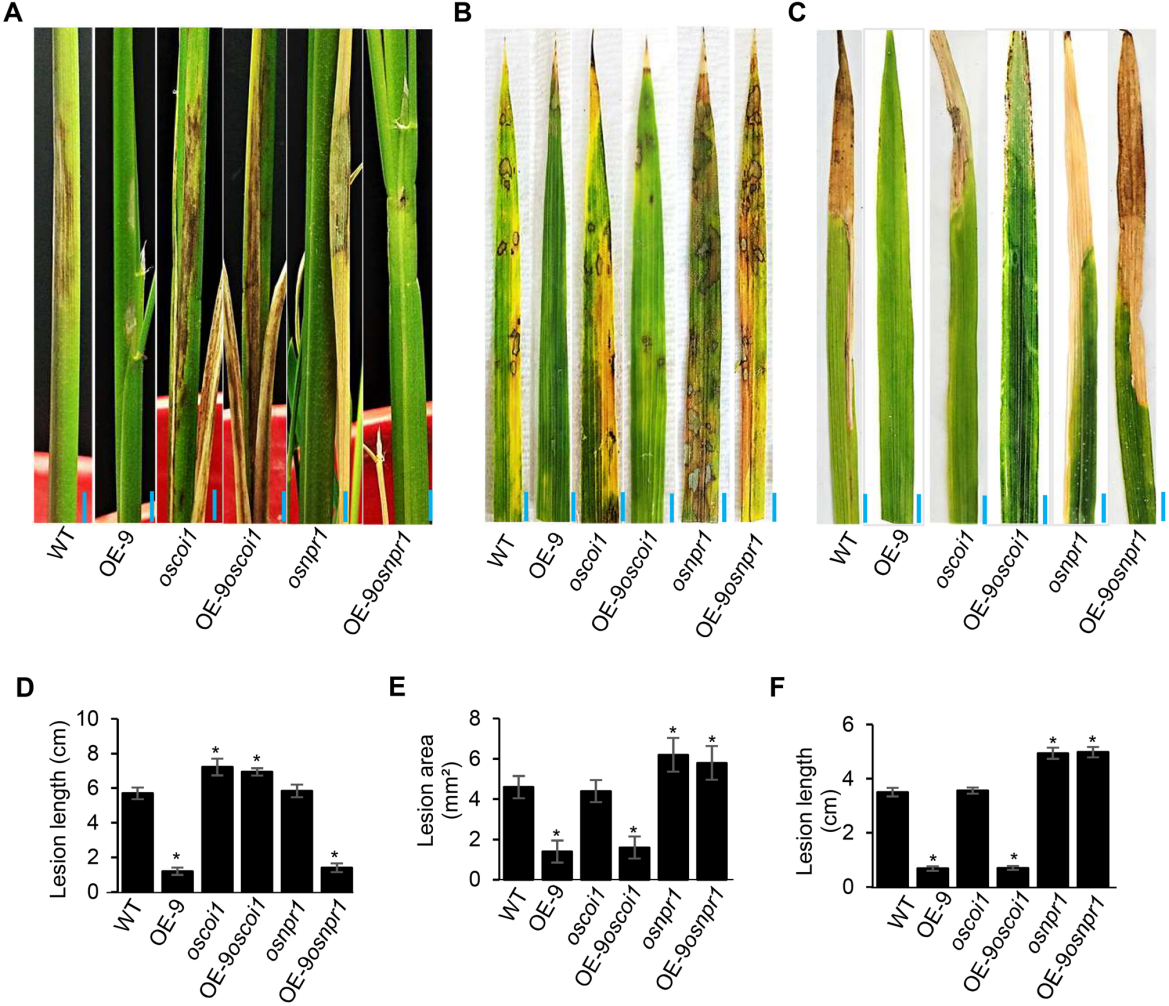
OsACBP5-OE transgenic rice plants showed protection against various plant pathogens via SA- and JA-induced defence responses. (**A**) Sheath blight symptoms after inoculation with the necrotroph *R. solani* on five-week-old WT, OsACBP5-OE line 9 (OE-9), JA-signalling deficient mutant *oscoi1, oscoi1* in the OE-9 background (OE-9*oscoi1*), SA-signalling deficient mutant *osnpr1* and *osnpr1* in the OE-9 background (OE-9*osnpr1*) at 14 days post-inoculation (dpi). Blue bars = 1 cm. (**B**) Rice blast symptoms on 3-week-old WT, *oscoi1,* OE-9*oscoi1, osnpr1* and OE-9*osnpr1* inoculated with the hemibiotrophic fungal pathogen, *M. oryzae* 7 dpi. Blue bars = 5 mm. (**C**) Disease phenotype in 3-week-old WT, *oscoi1,* OE-9*oscoi1, osnpr1* and OE-9*osnpr1* inoculated with the bacterial biotroph *Xoo* 14 dpi. Blue bars = 5 mm. (**D**) Lesion length following *R. solani* infection; (**E**) lesion area after inoculation with *M. oryzae*; (**F**) lesion length following *Xoo* infection in the WT, OE-9*, oscoi1,* OE-9*oscoi1, osnpr1* and OE-9*osnpr1*. Data points represent means ± SD from three independent experiments. Asterisks indicate significant difference (*P* < 0.05) in comparison to the controls by Student’s *t-*test.

To determine if SA- and JA-signalling pathways are involved in the enhanced resistance of rice OsACBP5-OEs against various plant pathogens, WT, OsACBP5-OE9 (OE-9)*, osnpr1* (SA-signalling-deficient mutant), *oscoi1* (JA-signalling-deficient mutant) and OE-9 in the *osnpr1* or *oscoi1* backgrounds were infected with the necrotroph *R. solani* (Fig. 5A)*,* the hemibiotroph *M. oryzae* (Fig. 5B) and the biotroph *Xoo* (Fig. 5C)*.* The measurement of lesion length in *R. solani-*infected plants showed significantly higher susceptibility in OE-9*oscoi1* and *oscoi1* plants in comparison to the WT (Fig. 5D). No significant difference was observed between *osnpr1* and the WT (Fig. 5D). The OE-9*osnpr1* mimicked the response of OE-9 (Fig. 5D). These results indicated that the improved resistance of OsACBP5-OEs to the fungal necrotroph *R. solani* is JA-dependent. When the plants (WT, OE-9*, osnpr1, oscoi1*, OE-9*osnpr1* and OE-9*oscoi1*) were infected with the hemibiotrophic fungal pathogen *M. oryzae* and the biotroph *Xoo,* OE-9*osnpr1* plants were no longer resistant to the pathogen similar to *osnpr1* (Fig. 5, E and F). No significant difference was observed in *M. oryzae-* or *Xoo-*infected WT and *oscoi1* mutant (Fig. 5E,F). OE-9 and OE-9*oscoi1* showed similar responses to representative hemibiotrophic (*M. oryzae*) and biotrophic (*Xoo*) pathogen infection (Fig. 5E,F). These findings illustrate that the SA-signalling pathway is responsible for the enhanced resistance of OsACBP5-OEs to infection caused by the representative hemibiotroph and biotroph.

Furthermore, GC-MS was performed to measure SA and JA content in *R. solani-, M. grisea-* and *Xoo-*infected WT, OE-9*, osnpr1, oscoi1*, OE-9*osnpr1* and OE-9*oscoi1. R. solani-*infected *oscoi1* and OE-9*oscoi1* respectively showed 40- and three-fold lower JA content compared to the WT (Supplemental Fig. S2A). When qRT-PCR was performed, *oscoi1* and OE-9*oscoi1* respectively showed ~ ten- and three-fold lower expression of *OsAOS1* in comparison to that of WT (Supplemental Fig. S3A). Similarly, *M. grisea-* and *Xoo-*infected *osnpr1* and OE-9*osnpr1* respectively showed ~ 20- and 2.5-fold lower SA content compared to the WT (Supplemental Fig. S2B,C). When qRT-PCR was performed, *osnpr1* and OE-9*osnpr1* respectively showed ~ ten- and three-fold lower expression of *OsNPR1* compared to the WT (Supplemental Fig. S3B,C). These results further confirm that the improved resistance of OsACBP5-OEs to the necrotroph *R. solani* is JA-dependent and enhanced resistance of OsACBP5-OEs to hemibiotroph (*M. grisea*) and biotroph (*Xoo*) is SA-dependent.

### Two W-boxes in the *OsACBP5* 5′-flanking region bind infected rice nuclear proteins

**Figure 6:**
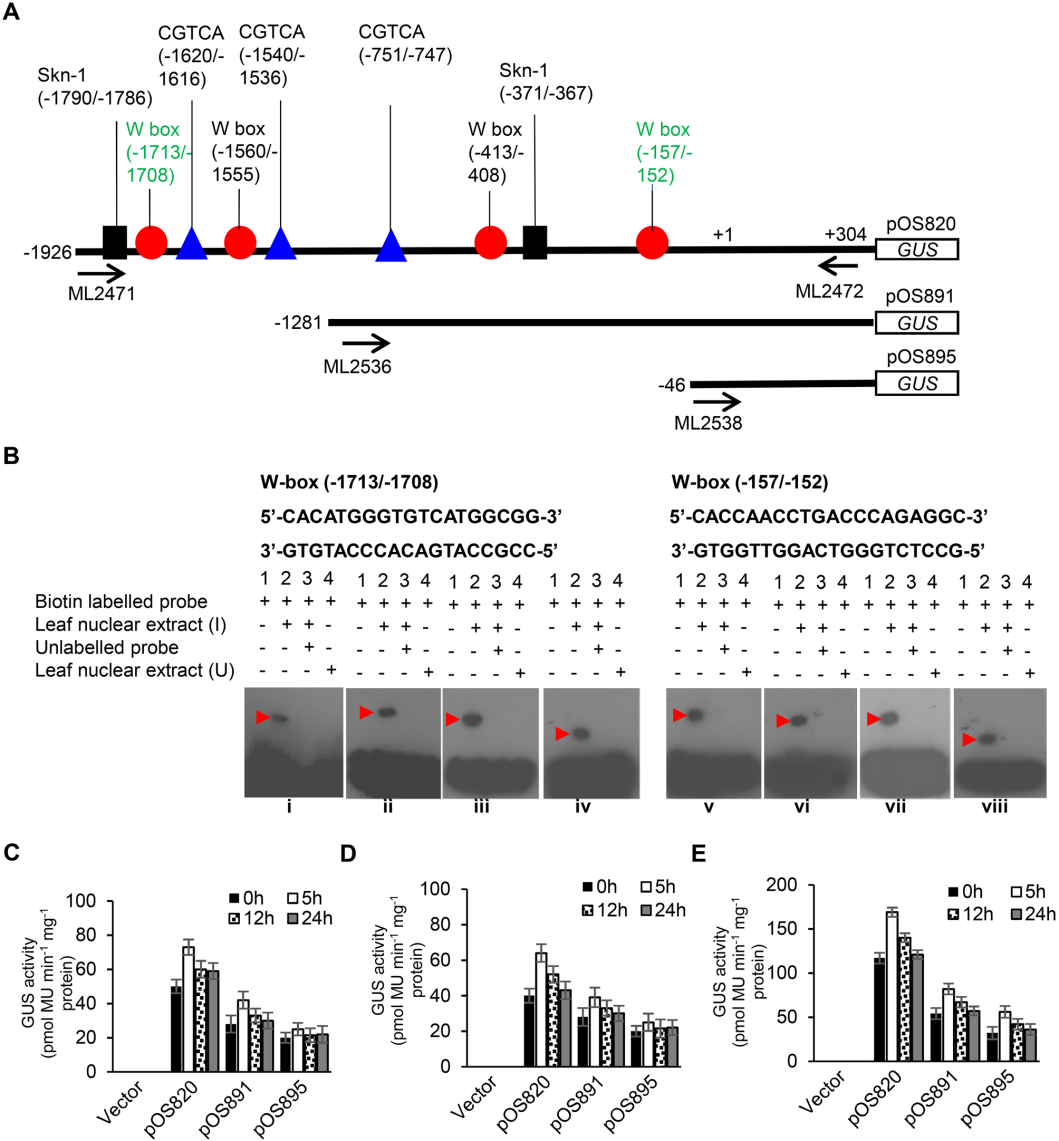
Analysis of the *OsACBP5* 5′-flanking region. (**A**) A schematic diagram of constructs (pOS820, pOS891 and pOS895) developed by 5′-end deletion of the *OsACBP5* 5′-flanking region (− 1926/ + 304). Promoter fragments of different lengths were introduced into the binary vector DX2181 comprising the *GUS* reporter gene. Black bars (not to scale) represent each truncated fragment and the end position of each deletion is denoted on the left. Putative *cis-*elements (in black, blue and red symbols are labelled) on the *OsACBP5pro::GUS* construct pOS820. The *cis-*elements labelled in green have been verified experimentally. Forward and reverse arrows indicate the PCR primers used for generating constructs. (**B**) Interaction of the infected WT rice leaf nuclear extract (I) with the W-box (− 1713/− 1708 and − 157/− 152) probe. Nucleotide sequences of double-stranded oligonucleotides used in EMSAs are shown in bold. The protein-DNA complexes are indicated by red arrowheads. Lane 1, free probe without the addition of leaf nuclear extracts. Crude nuclear extracts from infected leaves were incubated with biotin end-labelled probes (lane 2) in the presence of a 500-fold molar excess of an unlabelled competitor (lane 3). Lane 4, a negative control with labelled probe and untreated leaf nuclear extract (U). I, *R. solani-*infected nuclear extract in panels i and v; *M. oryzae-*infected nuclear extract in panels ii and vi; *Xoo-*infected nuclear extract in panels iii and vii and *C. oryzae*-infected nuclear extract in panels iv and viii. Quantitative fluorometric measurement of GUS activity in (**C**) SA-treated, (**D**) MeJA-treated and (**E**) *R. solani-*infected *OsACBP5pro::GUS* constructs pOS820, pOS891 and pOS895 0 h, 5 h, 12 h and 24 h post-treatment. Five independent lines were used per construct. Data points represent means ± SD from three independent experiments.

Given that OsACBP5 functions in plant defence, the 5′-flanking region of *OsACBP5* was investigated using the PlantCARE^62^ (https://bioinformatics.psb.ugent.be/webtools/plantcare/html/) and PLACE^63^ (https://www.dna.affrc.go.jp/PLACE/) databases. Potential *cis*-elements identified in the *OsACBP5* 5′-flanking region include pathogen-responsive *cis*-elements such as the W-box^64^ (− 1713/− 1708, − 1,560/− 1555, − 413/− 408 and − 157/− 152), MeJA-responsive element CGTCA^65^ (− 1,620/− 1616, − 1,540/− 1536 and − 751/− 747) and seed-specific motifs such as Skn-1^66^ (− 1,790/− 1786 and − 371/− 367) (Fig. 6A). EMSAs using crude nuclear extracts from *R. solani-*infected 5-week-old WT rice, *C. oryzae-*infected three-week-old WT rice, *M. oryzae-*infected three-week-old WT rice, *Xoo-*infected three-week-old WT rice and *C. oryzae-*infected three-week-old WT rice showed strong DNA–protein binding complexes with the W-boxes at − 1713/− 1708 and − 157/− 152 (Fig. 6B), indicating that two of the four putative W-boxes are essential in regulating *OsACBP5* expression. In contrast, when the CGTCA and Skn-1 boxes were tested, they did not bind to nuclear extracts in EMSA (Supplemental Fig. S4).

### *OsACBP5pro::GUS* expression is induced by SA, MeJA and *R. solani* infection

To identify the *cis-*elements of the *OsACBP5* 5′-flanking region in SA-, methyl jasmonate (MeJA)- and pathogen-induced regulation, quantitative GUS assays were performed on three-week-old (T_3_-generation) transgenic rice pOS820 (2.2-kb *OsACBP5pro::GUS*), pOS891 (1.3-kb *OsACBP5pro::GUS*) and pOS895 (0.6-kb *OsACBP5pro::GUS*) transformants. When SA- (100 µM) and MeJA- (100 µM) treated three-week-old rice seedlings were analysed 0 h, 5 h, 12 h and 24 h post-treatment, pOS820, pOS891 and pOS895 transformants showed higher GUS activity 5 h post-treatment (Fig. 6C,D). However, the pOS820 transformants showed 1.7-fold and 2.4-fold increased GUS activity 5 h post-SA treatment over the pOS891 and pOS895 transformants, respectively (Fig. 6C). Similarly, the pOS820 transformants showed 1.6-fold and 2.5-fold increased GUS activity 5 h post-MeJA treatment over the pOS891 and pOS895 transformants, respectively (Fig. 6D). Likewise, when *R. solani*-infected rice seedlings were analysed, the pOS820 transformants showed twofold and threefold increased GUS activity over the pOS891 and pOS895 transformants, respectively (Fig. 6E), demonstrating that *OsACBP5pro::GUS* expression in seedlings was induced by SA, MeJA and *R. solani* treatment. Reduction in GUS activity in the pOS891 and pOS895 transformants suggested that the W-boxes (− 1713/− 1708 and − 157/− 152) play an important role in the regulation of *OsACBP5*.

### Recombinant OsACBP5 binds 18:3-acyl-CoA ester

Lipidex assays by Meng et al*.* have shown the binding of (His)_6_-tagged OsACBP5 to 18:3-acyl-CoA esters^28^. As 18:3-FA is important for basal defence against fungal pathogens and is a precursor for JA biosynthesis^67^, the binding affinity of (His)_6_-OsACBP5 to 18:3-acyl-CoA ester was investigated by isothermal titration calorimetry (ITC) which provides a more precise method to measure protein-ligand binding than Lipidex assays^20^. Consistent with Lipidex assays, recombinant OsACBP5 (rOsACBP5) was shown to bind to 18:3-acyl-CoA with high affinities (Supplemental Fig. S5). ITC results (Supplemental Table S1) indicated that rOsACBP5 has a strong binding affinity to 18:3-acyl-CoA ester with a dissociation constant (*K*_d_) value of 59.5 nM.

When OsACBP5-OE leaves were further examined using GC-MS to test the level of the six major FA species (14:0-, 16:0-, 18:1-, 18:2-, 18:3- and 20:0-FAs), the three most abundant species were 16:0-, 18:2- and 18:3-FAs (Supplemental Fig. S6). OsACBP5-OEs (OE-1, OE-3, OE-6, OE-9 and OE-11) showed twofold higher 18:3-FA content in leaves than the wild-type and vector-transformed controls (Fig. S6). However, no significant differences were detected for 14:0-, 16:0-, 18:1-, 18:2-, and 20:0-FAs between the OsACBP5-OEs and the controls (Fig. S6).

### Rice genes are differentially expressed between OsACBP5-OEs and the wild type in response to *R. solani* infection

**Figure 7:**
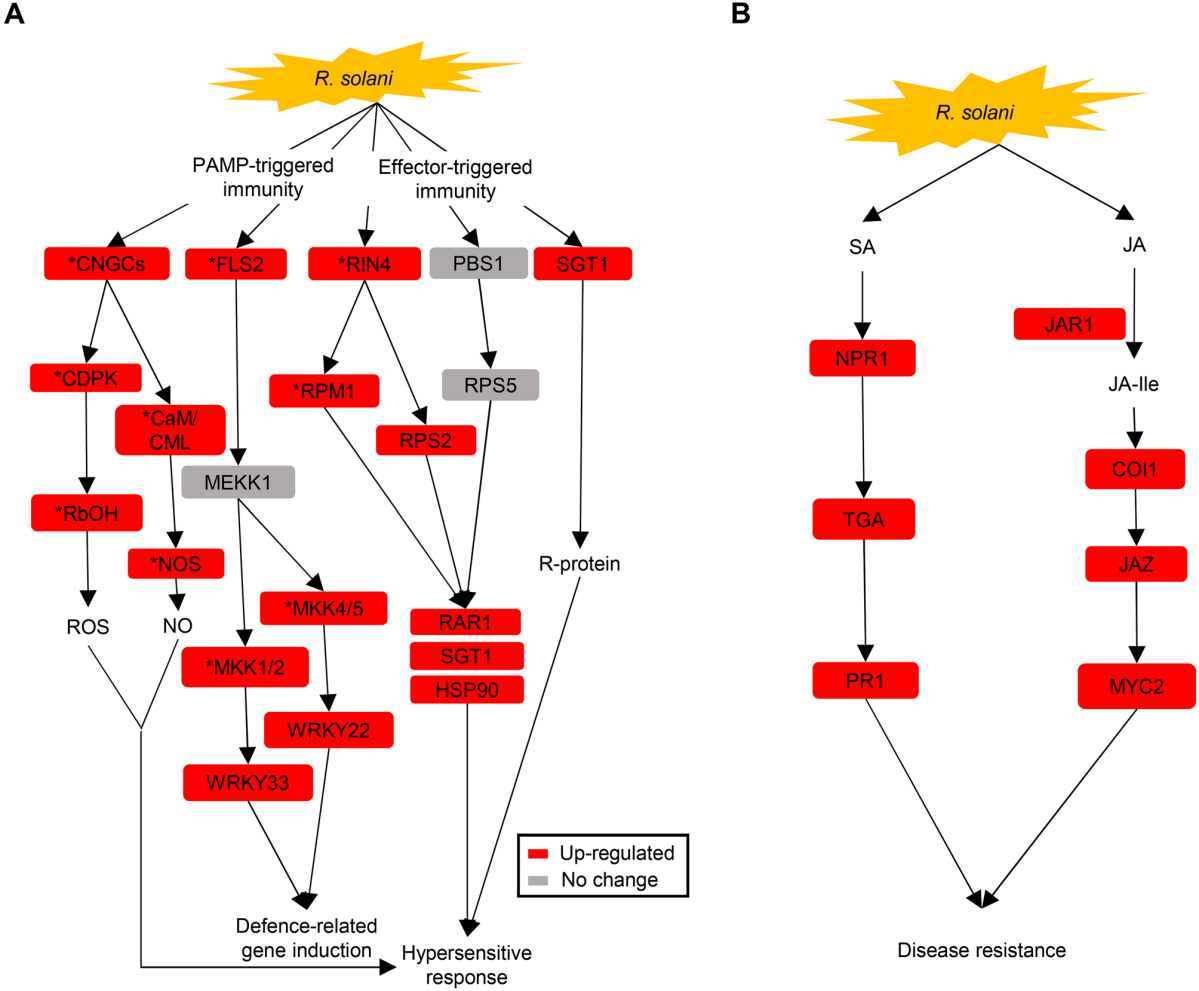
DEGs associated with the plant-pathogen interaction pathway as well as SA- and JA-signalling pathways in *R. solani*-infected OsACBP5-OEs. The KEGG database^148–150^ was used for pathway analysis. (**A**) Increase in the cytosolic Ca^2+^ concentration by the activation of *CNGCs*, *CDPK* and *CaM/CML* in PTI, is a regulator for production of ROS and NOS which results in the hypersensitive response^121,122^. Activation of *FLS2* in PTI triggers the MAPK signalling pathway that induces known defence genes for the generation of antimicrobial compounds such as phytoalexins, camalexin and lignin^123,124^. Pathogen infection induces *RIN4* in ETI, which activates the disease resistant proteins RPM1 and RPS2^92^. RPM1 and RPS2 then trigger a complex formed by *HSP90, RAR1* and *SGT1* leading to the hypersensitive response^96–98^. *SGT1* also regulates early *R* gene-mediated plant defences upon pathogen infection^125^. Up-regulated genes are boxed in red, genes those are not affected are boxed in grey. Those marked with asterisks in this figure have been previously discussed^126^. (**B**) SA triggers the accumulation NPR1 which activates the *TGA* transcription factor and *PR1* resulting in plant defence^127,128^. Activation of *JAR1* following pathogen infection catalyses the production of JA-Ile from JA. Production of JA-Ile is crucial for the JA-signalling pathway involving *COI1, JAZ* and *MYC2* leading to plant defence^47,129^. Up-regulated genes are boxed in red.

When transcriptomic analysis was performed on *R. solani*-infected transgenic rice OsACBP5-OEs, a total of 22,063 (15,253 up-regulated and 6,810 down-regulated) differentially expressed genes (DEGs) were identified between OsACBP5-OEs and the wild type control. Sixteen genes upregulated in the plant-pathogen interaction pathway (Kyoto Encyclopedia of Genes and Genomes (KEGG) map 04626) in OsACBP5-OEs following *R. solani* infection were *CYCLIC NUCLEOTIDE GATED CHANNELS (CNGCS), CALCIUM-DEPENDENT PROTEIN KINASE (CDPK), CALMODULIN/CALMODULIN-LIKE PROTEINS (CAM/CML), RESPIRATORY BURST OXIDASE HOMOLOG (RBOH), NITRIC OXIDE SYNTHASE (NOS), FLAGELLIN-SENSING2 (FLS2), MITOGEN-ACTIVATED PROTEIN KINASE KINASE1/2 (MKK1/2), MITOGEN-ACTIVATED PROTEIN KINASE KINASE4/5 (MKK4/5), WRKY TRANSCRIPTION FACTOR22 (WRKY22), WRKY TRANSCRIPTION FACTOR33 (WRKY33), DISEASE RESISTANT PROTEINS (RPM1, RPS2, RAR1), RPM1-INTERACTING PROTEIN4 (RIN4), SUPPRESSOR OF G2 ALLELE OF SKP1 (SGT1)* and *HEAT SHOCK PROTEIN90 (HSP90)* (Fig. 7A). Ten DEGs related to the PAMP-triggered immunity (PTI) signalling pathway include those encoding CNGCs, CDPK, CaM/CML, RbOH, NOS, FLS2, MKK1/2, MKK4/5, WRKY22 and WRKY33 (Fig. 7A). The six DEGs upregulated in the effector-triggered immunity (ETI) signalling pathway were *RPM1, RPS2, RAR1, RIN4, SGT1* and *HSP90* (Fig. 7A).

Furthermore, seven DEGs related to the SA- and JA-signalling pathways were up-regulated in *R. solani* infected OsACBP5-OEs (Fig. 7B). In the JA signalling pathway, four DEGs encoding JASMONOYL ISOLEUCINE CONJUGATE SYNTHASE1 (JAR1), CORONATINE INSENSITIVE PROTEIN1 (COI1), JASMONATE ZIM-DOMAIN CONTAINING PROTEIN (JAZ) and TRANSCRIPTION FACTOR MYC2 were induced upon fungal infection (Fig. 7B). Similarly, three DEGs in the SA-signalling pathway such as *NON-EXPRESSOR OF PATHOGENESIS-RELATED1 (NPR1)*, *TRANSCRIPTION FACTOR TGA* and *PATHOGENESIS-RELATED PROTEIN1 (PR1)* were up-regulated following pathogen invasion of OsACBP5-OEs leading to disease resistance (Fig. 7B). Table S2 shows fold changes of DEGs associated with the plant-pathogen interaction pathway as well as SA- and JA-signalling pathways in *R. solani*-infected OsACBP5-OEs.

### Biotic stress-related proteins were induced in OsACBP5-OEs by *R. solani* infection

When SWATH-MS quantitative proteomic analysis was carried out to explore the effect of OsACBP5 action on *R. solani* infection, ProteinPilot software identified 1,365 proteins, 2,754 peptides, and 12,390 spectra with 99% confidence and 1% global false discovery rate (FDR). Of 1,365 identified proteins, 419 were significantly upregulated in rice OsACBP5-OEs versus WT and vector-transformed plants (*P* < 0.05). Consistent with transcriptomics data, proteins involved in the plant-pathogen interaction pathway (CDPK, FLS2, RPM1, RPS2 and HSP90), JA-signalling pathway (JAZ and MYC2) and SA-signalling pathway (PR1) were upregulated following *R. solani* infection in OsACBP5-OEs (Table 1).

**Table 1:** Biotic stress-related proteins with increased abundance in response to *R. solani* infection in transgenic rice OsACBP5-OEs.

	Accession	Protein name	Fold-change	*p*-value
Plant-pathogen interaction pathway	Os08g0540400	CALCIUM-DEPENDENT PROTEIN KINASE (CDPK)	3.40	0.004
	Os04g0618700	FLAGELLIN-SENSING2 (FLS2)	2.57	0.015
	Os07g0186500	DISEASE RESISTANT PROTEIN RPM1	3.02	0.024
	Os01g0788500	DISEASE RESISTANT PROTEIN RPS2	3.18	0.009
	Os08g0487800	HEAT SHOCK PROTEIN90 (HSP90)	2.87	0.016
JA signalling pathway	Os03g0180800	JASMONATE ZIM-DOMAIN CONTAINING PROTEIN (JAZ)	2.72	0.009
	Os10g0575000	TRANSCRIPTION FACTOR MYC2	3.35	0.001
SA signalling pathway	Os01g0382000	PATHOGENESIS-RELATED PROTEIN1 (PR1)	3.14	0.002

Proteins with fold change > 2.0 are considered upregulated.

When qRT-PCR was performed on OsACBP5-OEs to validate the results from transcriptomic and proteomic analyses, increased expression of genes involved in the plant-pathogen interaction pathway (*CNGCs, CDPK, CaM/CML, RbOH, NOS, FLS2, MKK1/2, MKK4/5, WRKY22, WRKY33, RPM1, RPS2, RAR1, RIN4, SGT1* and *HSP90*), JA-signalling pathway (*JAR1, COI1, JAZ* and *MYC2*) and SA-signalling pathway (*NPR1, TGA* and *PR1*) in OsACBP5-OEs (Supplemental Figs. S7–S9) supported the transcriptomic and proteomic data. The expression level of *ACTIN* in *R. solani-*infected WT, VC and OsACBP5-OEs is shown in Supplemental Figure S10. Taken together, findings from transcriptomics and proteomics suggest that OsACBP5 plays a crucial role in the protection of plants against *R. solani* through activating plant-pathogen interaction pathway as well as JA- and SA-signalling pathways.

## Discussion

### OsACBP5 conferred broad-spectrum defence against phytopathogens

In this study, the function of OsACBP5 in plant defence was established by phenotypic analyses of five independent rice OsACBP5-OE lines in response to representative necrotrophic, hemibiotrophic and biotrophic pathogens. Previous work on transgenic Arabidopsis overexpressing its homologue, AtACBP3, had shown that AtACBP3 could confer NONEXPRESSOR OF PR GENES1 (NPR1)-dependent resistance to bacterial biotroph *P. syringae,* with increased susceptibility to the fungal necrotroph *B. cinerea*^17^. In contrast, this study revealed that transgenic rice OsACBP5-OEs displayed enhanced tolerance to necrotrophic fungal pathogens such as *R. solani* and *C. oryzae* (Fig. 1)*,* hemibiotrophic fungal pathogens, *M. oryzae* and *F. graminearum* (Fig. 2) and a biotrophic bacterial pathogen, *Xoo* (Fig. 3). These findings demonstrated that OsACBP5 is more versatile against pathogens in transgenic rice. As Takato et al*.* had earlier illustrated that the overexpression of a Class III ACBP from grape (*Vitis vinifera*) could protect transgenic Arabidopsis against the biotroph *P. syringae* and a hemibiotroph *C. higginsianum*^30^*,* it appears that the Class III ACBPs are promising targets for disease prevention in both transgenic dicots and monocots. Similar to OsACBP5 in exhibiting broad-spectrum properties in defence, wide-range protection against *R. solani, M. oryzae* and *Xoo* have been reported in transgenic rice overexpressing a cysteine-rich antimicrobial defensin from *Allium cepa* (Ace-AMP), but the molecular regulation on its action is less understood^68^. In comparison, defence-related proteins such as the rice wall-associated kinase (OsWAK25) and MoSM1, encoding a cerato-platanin protein from *M. oryzae,* when overexpressed in rice, conferred protection only against the hemibiotroph *M. oryzae* and the bacterial biotroph *Xoo*, but displayed increased susceptibility to the fungal necrotroph *R. solani*^57,69^. Correspondingly, the expression of *OsWRKY13,* encoding transcription factor WRKY, was induced by *M. oryzae* and *Xoo* infection^58,86^. The constitutive expression of *OsWRKY13* displayed protection to *M. oryzae* and *Xoo *via the SA-signalling pathway^58,89^. Likewise, *M. oryzae* and *Xoo* infection induced the expression of rice *DEFENCE-RESPONSE PROTEIN8* (*OsDR8*), encoding a protein involved in thiamine biosynthesis^58,86^. OsDR8 accumulates thiamine and confers systemic acquired resistance (SAR) against *M. oryzae* and *Xoo*^58,140^. Similarly, the constitutive expression of rice *INDOLE-3-ACETIC ACID (IAA) AMIDO SYNTHETASE (GH3-8)*, whose expression is induced by auxin, enhanced resistance to *M. oryzae* and *Xoo* infection in rice by suppressing pathogen-induced IAA accumulation^58,141^. Other rice genes that promote similar pathogen resistance are summarised in Table 2.

**Table 2:** Reported rice genes conferring pathogen resistance.

Gene	Disease	Pathogen	Immune response	References
*SOMATIC EMBRYOGENESIS RECEPTOR KINASE2 (OsSERK2)*	Bacterial blight	*Xoo*	PAMP-triggered immunity	Ref.^142^
	Blast	*M. oryzae*		Ref.^143^
*WALL-ASSOCIATED KINASE25 (OsWAK25)*	Bacterial blight	*Xoo*	PAMP-triggered immunity	Ref.^144^
	Blast	*M. oryzae*	SA-mediated pathways	Ref.^69^
*DISEASE RESISTANT PROTEINS RAR1 (OsRAR1)*	Bacterial blight	*Xoo*	Effector-triggered immunity	Ref.^93^
	Blast	*M. oryzae*		
*WRKY TRANSCRIPTION FACTOR13 (OsWRKY13)*	Bacterial blight	*Xoo*	SA-signalling pathway	Ref.^89^
	Blast	*M. oryzae*		
*DEFENCE-RESPONSE PROTEIN8 (OsDR8)*	Bacterial blight	*Xoo*	Systemic acquired resistance	Ref.^140^
	Blast	*M. oryzae*		
*IAA-AMIDO SYNTHETASE (GH3-8)*	Bacterial blight	*Xoo*	Suppressing pathogen-induced IAA accumulation	Ref.^141^
	Blast	*M. oryzae*		
*GERMIN-LIKE PROTEIN (OsGLP2-1)*	Bacterial blight	*Xoo*	Accumulation of hydrogen peroxide and JA	Ref.^145^
	Blast	*M. oryzae*		
*NPR1 HOMOLOGUE (OsNH1)*	Bacterial blight	*Xoo*	Systemic acquired resistance	Ref.^147^
	Blast	*M. oryzae*		
*LYSIN MOTIF–CONTAINING PROTEINS (OsLYP)*	Bacterial blight	*Xoo*	PAMP-triggered immunity	Ref.^146^
	Blast	*M. oryzae*		
*WRKY TRANSCRIPTION FACTOR30 (OsWRKY30)*	Sheath blight	*R. solani*	JA-mediated defence	Ref.^90^
	Blast	*M. oryzae*		

### Rice OsACBP5-OE*s* showed JA-mediated response against necrotrophs and SA-mediated response against (hemi)biotrophs

SA and JA, the two critical defence signalling hormones that play vital roles against necrotrophic, hemibiotrophic and biotrophic pathogens in rice^61,70^, were observed to accumulate in rice OsACBP5-OEs (Fig. 4A,B). The upregulated expression of *OsNPR1,* an SA-signalling regulatory gene, and *OsAOS2* encoding allene oxide synthase in JA biosynthesis, in OsACBP5-OEs (Fig. 4C,D) likely stimulates SA- and JA-mediated defence responses. Furthermore, results from bioassays on transgenic rice OsACBP5-OE9 in *oscoi1* and *osnpr1* backgrounds suggest that necrotrophic resistance in rice OsACBP5-OEs is JA-dependent and (hemi)biotrophic resistance is SA-dependent (Fig. 5). A recent study has shown that transgenic Arabidopsis overexpressing OsACBP5 were conferred resistance to necrotrophic (*R. solani, B. cinerea, A. brassicicola*), hemibiotrophic (*C. siamense*) and biotrophic (*P. syringae*) phytopathogens^59,60^. Proteomic analysis on the *R. solani-*infected transgenic Arabidopsis OsACBP-OEs showed upregulation of biotic stress-related proteins including cell wall-related proteins such as FASCILIN-LIKE ARABINOGALACTAN-PROTEIN10, LEUCINE-RICH REPEAT EXTENSIN-LIKE PROTEINS, XYLOGLUCAN ENDOTRANSGLUCOSYLASE/HYDROLASE PROTEIN4 and PECTINESTERASE INHIBITOR18; proteins associated with glucosinolate degradation including GDSL-LIKE LIPASE23, EPITHIOSPECIFIER MODIFIER1, MYROSINASE1, MYROSINASE2 and NITRILASE1; as well as a protein involved in jasmonate biosynthesis, ALLENE OXIDE CYCLASE2^59,60^. These results from proteomic analysis indicated that the defence responses arising from OsACBP5 overexpression in transgenic Arabidopsis involved cell wall-mediated defence as well as salicylic acid (SA)- and jasmonic acid (JA)-mediated defence pathways^59,60^. Similarly, transgenic Arabidopsis overexpressing AtACBP3 displayed enhanced SA-mediated resistance to the biotrophic pathogen *P. syringae*^17^.

The current results on transgenic rice OsACBP5-OEs also demonstrated the cooperation between the SA and JA pathways in defence against representative necrotrophs (*R. solani, C. oryzae*)*,* hemibiotrophs (*M. oryzae, F. graminearum*) and biotrophs (*Xoo*). In contrast, the SA and JA defence signalling pathways generally interact antagonistically in dicots^51,53,55,71–78^, while such interaction is not well investigated in monocots such as rice^56,79^. Nonetheless, Tamaoki et al*.* reported that SA and JA can collaboratively stimulate a common defence signalling system in rice against pathogens^80^. Similar to the present study, transgenic MoSM1-OE rice displayed improved resistance to the hemibiotroph *M. oryzae* and the biotroph *Xoo* accompanied by elevated SA and JA content and upregulated expression of SA- and JA-signalling genes^57^. SA and JA accumulation in rice OsACBP5-OEs may have arisen from the ability of OsACBP5 in binding to 18:3-acyl-CoA ester because ITC data supported rOsACBP5 binding to 18:3-acyl-CoA ester with a *K*_d_ value in the nanomolar range (Fig. S5). Interestingly, OsACBP5-OEs showed higher linolenic acid (18:3) content than the controls (Fig. S6) and 18:3-FA is a precursor for JA biosynthesis^67^. Previous reports have demonstrated that the *Arabidopsis* *ssi2* mutant contains lower 18:3-FA content than the WT and was more susceptible to necrotroph *B. cinerea* infection^82^. These results resonate well with the current study which revealed that decreased susceptibility of OsACBP5-OEs to various pathogens, in comparison to the WT, is associated with increase in 18:3-FA content.

The physiological significance in the role of AtACBP3 in trafficking lipids such as acyl-CoA esters was evident in transgenic Arabidopsis overexpressing AtACBP3 which displayed accelerated leaf senescence, in contrast to an *atacbp3* T-DNA insertional mutant and AtACBP3 RNA interference (RNAi) transgenic Arabidopsis lines which were delayed in dark-induced leaf senescence^15^. Subsequent acyl-CoA and lipid profiling revealed that AtACBP3 overexpression culminated in the accumulation of acyl-CoA and phosphatidylethanolamine (PE), while the downregulation of AtACBP3 reduced PE^15^. In dark-treated and premature senescing AtACBP3-OE plants, PC and phosphatidylinositol levels declined accompanied by increases in PA, lysophospholipids, and oxylipin-containing galactolipids (arabidopsides). It was concluded that the accumulation of PA and arabidopsides (A, B, D, E, and G) resulting from lipid peroxidation in AtACBP3-OEs likely caused leaf senescence^15^. In another study, it was reported that oxylipin-related FA (18:2-FA, 18:3-FA and MeJA) content was lower in *atacbp3* and *AtACBP3*-RNAi than wild-type phloem exudates upon GC-MS analysis^28^. On ITC analysis, recombinant AtACBP3 was shown to bind medium- and long-chain acyl-CoA esters with K_*D*_ values in the micromolar range^28^. Hu et al*.* concluded that the phloem-mobile AtACBP3 likely affected the FA pool and JA content in the phloem by its binding to acyl-CoA esters, ultimately influencing the level of oxylipins, which are crucial components of the plant wound responses mobilized via the vasculature^28^.

### Significance of W-boxes in regulating pathogen-inducible *OsACBP5* expression

The WRKY family of TFs that regulate the transcription of plant defence genes through the W-box^84^, are crucial in protection against necrotrophic, hemibiotrophic and biotrophic pathogens^84–94^. For example, OsWRKY4 binds to the W-boxes in the 5′-flanking region in each of pathogenesis-related *PR1b* and *PR5,* and *OsWRKY4* and *OsWRKY80* were reported to be highly induced by *R. solani* infection^91,95^. Wang et al*.* also showed that transgenic rice overexpressing OsWRKY4 were protected against *R. solani* infection^95^. In this study, OsACBP5-OEs were proven tolerant to representative necrotrophs, hemibiotrophs and biotrophic phytopathogens and EMSAs revealed that only two of the four W-boxes (-1713/-1708 and -157/-152) in the 5′-flanking region of *OsACBP5* regulate *OsACBP5* expression during representative necrotrophic (Fig. 6B panels i, iv, v and viii), hemibiotrophic (Fig. 6B panels ii and vi) and biotrophic (Fig. 6B panels iii and vii) infection. These results correspond well with quantitative GUS assays on the pOS820 (2.2-kb *OsACBP5pro::GUS;* -1926/ + 304) transformants which displayed induced GUS expression after treatment with the pathogen-related phytohormones, SA and MeJA, in comparison to transformants from constructs that lacked either of these W-boxes, pOS891 (1.3-kb *OsACBP5pro::GUS;* − 1,281/ + 304) and pOS895 (0.6-kb *OsACBP5pro::GUS;* − 46/ + 304) (Fig. 6C,D). These results verified that the two W-boxes (− 1713/− 1708 and − 157/− 152) play a role in regulating *OsACBP5* expression in response to necrotrophic, hemibiotrophic and biotrophic pathogens, as well as to the pathogen-related phytohormones, SA and MeJA.

Previous results suggest that increased protection to *R. solani* and *M. oryzae* in transgenic rice overexpressing OsWRKY30 was associated with elevated levels of JA, as well as the stimulated expression of JA synthesis-related genes (*LOX* and *AOS2*) and pathogenesis-related *PR3* and *PR10,* following fungal pathogen infection^90^. Furthermore, Hiroyuki and Terauchi revealed that the W-boxes in the *RICE THAUMATIN-LIKE PROTEIN1* (*RTLP1*) promoter function in response to *M. oryzae* infection^96^. Also, past investigations on the development of resistance in rice against the rice blast pathogen *M. oryzae* unveiled a critical role for WRKY TFs (OsWRKY45, OsWRKY13 and OsWRKY42) in plant defence^92^. OsWRKY45 has been assigned a vital role in SA-mediated signalling in rice against the hemibiotrophic pathogen *M. oryzae*^84^. Enhanced resistance of transgenic rice to the biotrophic pathogen *Xoo* and the hemibiotroph *M. oryzae* was achieved by OsWRKY13 overexpression that was related to activation of SA-signalling and suppression of the JA-dependent pathway^89^. Similar to *OsACBP5,* where W-boxes were observed to bind nuclear extracts from *Xoo-*infected rice, W-boxes in rice *STRESS RESPONSIVE NAC1* (*SNAC1*) interacted with *Xoo*-treated nuclear proteins and OsWRKY13 was subsequently identified to regulate *SNAC1* expression during biotic stress^97^. Furthermore, the overexpression of OsWRKY13 or OsWRKY71 culminated in better tolerance to *Xoo* in transgenic rice^87,98^. Taken together these studies support a role for OsWRKY TFs in biotrophic, hemibiotrophic and necrotrophic fungal tolerance in rice via the SA- and JA-defence signalling pathways.

### Several defence-related genes were upregulated in *R. solani-*infected OsACBP5-OEs

The role of SA and JA in hemi(bio)trophic and necrotrophic pathogen defence in transgenic rice OsACBP5-OEs was partially confirmed from pathogen assays, GC-MS and qRT-PCR. Transcriptomic and proteomic analyses further confirmed the mechanism of defence in transgenic rice OsACBP5-OEs. Although transcriptomic and proteomic assays were performed only on necrotrophic pathogen *R. solani-*infected transgenic rice OsACBP5-OEs, the upregulated genes and proteins from these assays were reported to be involved in defence against necrotrophic, hemibiotrophic and biotrophic phytopathogens, which are discussed in this section.

Transcriptomics and proteomics data provided an insight into the defence responses of transgenic rice OsACBP5-OEs to the necrotrophic pathogen *R. solani* infection. The innate immunity in plants appeared to be triggered through PTI followed by ETI, providing the first line of defence upon pathogen challenge^99,100^. Ten genes involved in PTI were up-regulated in OsACBP5-OEs upon *R. solani* infection (Fig. 7A). Cytoplasmic Ca^2^^+^ concentration increases during PTI leading to the activation of CDPK in plant cells^101^. In this study, three genes involved in Ca^2+^ signalling were up-regulated in *R. solani-*infected OsACBP5-OEs including CNGCs, CDPK, CaM/CML. Calcium signalling was reportedly accompanied by an increase of both ROS and NO leading to SA-mediated defence^30,101^. Transcription factors WRKY22 and WRKY33 were activated by components of the MAPK cascade such as MEKK1, MKK1/2 and MKK4/5, resulting in induced expression of defence-related genes in *R. solani-*infected OsACBP5-OEs (Fig. 7A). Similar results were observed in *Xoo-*infected rice plants in which FLS2 perceived bacterial flagellin and activated the MAPK cascade which in turn activated WRKY22 and WRKY33 resulting in induced expression of defence-related genes^92,99,100^. Taken together, ROS production and activation of MAPKs and CDPKs cause an array of defences restricting pathogen progression.

In plants, a secondary immune response ETI is the basis for a second layer of defence^99,100^. The second signalling pathway consists of five genes encoding receptor proteins (RIN4, PBS1, RPM1, SGT1 and RAR1) to perceive pathogen infection. In this study, four such genes encoding receptor proteins including RIN4, RPM1, SGT1 and RAR1 displayed up-regulation in OsACBP5-OEs following *R. solani* infection (Fig. 7A). RPM1 recognizes modifications of RIN4 followed by *P. syringae* infection in Arabidopsis and RPM1 interacts with RIN4 triggering RPM1-mediated immunity^102^. RAR1 and SGT1 conferred resistance against *Xoo* and *M. oryzae* when overexpressed in rice^103,104^. RAR1 forms a complex with the molecular chaperones HSP90 and SGT1 to initiate a signalling cascade in diverse plant immune responses^105–108^. In this study, the upregulated expression of various components (HSP90, RAR1 and SGT1) of the complex likely caused a hypersensitive response in OsACBP5-OEs following *R. solani* infection (Fig. 7A). Previous studies have reported that hypersensitive responses are mostly accompanied by an increase in SA biosynthesis^109,110^.

Furthermore, several DEGs (*NPR1, TGA, PR1, JAR1, COI1, JAZ* and *MYC2*) involved in the SA- and JA-signalling pathways were enriched in *R. solani-*infected OsACBP5-OEs (Fig. 7B), suggesting that both pathways are involved (Fig. 7B), supporting the role of SA and JA against *R. solani*.

## Conclusions

The present study demonstrates that OsACBP5 is effective and activates defence responses in transgenic rice against various representative necrotrophic, hemibiotrophic and biotrophic pathogens. Transgenic rice OsACBP5-OEs showed higher SA and JA levels when compared to the WT and vector-transformed control, suggesting that both SA- and JA-mediated signalling pathways are activated in OsACBP5-OEs. These results demonstrate that OsACBP5 overexpression in rice effectively conferred broad-spectrum resistance against several phytopathogens (*R. solani, C. oryzae, M. oryzae, F. graminearum* and *Xoo*), providing a potential for OsACBP5 in enhancing disease resistance in crop plants.

## Methods

### Plant materials and growth conditions

T-DNA insertion mutants *osnpr1* and *oscoi1* were purchased from Rice T-DNA Insertion Sequence Database (RISD DB; cbi.khu.ac.kr/RISD_DB.html). Plasmid vectors, pCAMBIA1304 and DX2181, were obtained from Shanghai Normal University and Huazhong Agricultural University, respectively. T_3_-generation seeds of transgenic rice derived from this study including vector-transformed controls (pCAMBIA1304 and DX2181), OsACBP5-OEs, *OsACBP5pro::GUS,* OsACBP5-OE9*osnpr1* and OsACBP5-OE9*oscoi1* as well as *osnpr1, oscoi1,* and *Oryza sativa* cv Zhonghua11 wild-type (WT) seeds (ten seeds were used for each line per experiment) were surface-sterilized with 70% ethanol for 5 min followed by 3% sodium hypochlorite solution for 40 min. The seeds were then washed with distilled water 5 times and germinated on half-strength MS^115^ medium containing 3% sucrose for 1 week at 28 °C. One-week-old seedlings (five seedlings for each line per experiment) were transferred to clay soil in separate pots in a growth chamber under a 12 h light (28 °C)/12 h dark (25 °C) photoperiod^116^. Supporting Information provides details on the generation of transgenic rice (OsACBP5-OEs, OsACBP5-OE9*osnpr1,* OsACBP5-OE9*oscoi1, OsACBP5pro::GUS* fusion and its deletion derivatives), pathogen assays, phytohormone treatments, fluorometric assays of GUS activity, electrophoretic mobility shift assays (EMSAs), isothermal titration calorimetry (ITC) experiments and Quantitative Real Time-Polymerase Chain Reaction (qRT-PCR).

### Quantification of SA and JA

SA and JA quantification was performed following Fina et al*.*^116^. Leaf tissue (300 mg) was homogenized and SA extracted in 80% methanol by shaking for 16 h at − 20 °C. The samples were then purified on a C18 cartridge (Bond Elut C18 6 cc, 500 mg, Agilent, CA, USA) in 80% methanol. Formic acid was added for the binding of SA to the cartridge. The SA was eluted with diethyl ether. The eluent was evaporated under nitrogen gas after removing the residual water. The sample was further methylated using diazomethane and dried under nitrogen gas. The sample was subsequently dissolved in 100% hexane for GC-MS analysis. The same protocol was followed for JA quantification. SA (10 µM) and JA (10 µM) were used as internal standards.

### Expression and purification of OsACBP5

The (His)_6_-OsACBP5 recombinant protein was expressed in the soluble fraction of *Escherichia coli* BL21(DE3) Star pLysS (Invitrogen) cells transformed with plasmid pOS543, derived from vector pRSETA (Life Technologies) following Meng et al*.* (2011). (His)_6_-OsACBP5 was purified by using a HisTrap HP column (GE Healthcare) charged with 0.1 M NiCl_2_ according to Guo et al*.*^117^.

### Transcriptome analysis

Total RNA was extracted from *R. solani*-infected WT, vector (pCAMBIA1304)-transformed control and transgenic rice OsACBP5-OEs using the RNeasy Plant Mini Kit (Qiagen). RNAs samples were sequenced using BGISEQ-500 sequencer at Beijing Genomics Institute (BGI, Hong Kong). RNA concentration and quality were measured using Agilent 2100 Bio analyser (Agilent RNA 6000 Nano Kit). The BGISEQ-500 platform was used to sequence the cDNA libraries. SOAPnuke was used to filter reads and after filtering, the clean reads were stored in FASTQ format. The clean reads were mapped using Bowtie2 (https://bowtie-bio.sourceforge.net/Bowtie2/index.shtml) and the gene expression level was calculated using RSEM (https://deweylab.biostat.wisc.edu/RSEM). Differentially expressed genes (DEGs) were detected using DEGseq software based on the Poisson distribution. The KEGG database^148–150^ was used for pathway analysis. The open reading frame (ORF) of each DEG was identified using GETORF database. To predict the transcription factor of each DEG, the ORF was aligned to transcription factor domains using the PLNTFdb database. DEGs were mapped to the PRGdb database using BLAST to detect plant disease resistance genes.

### Sequential window acquisition of all theoretical mass spectra quantitative proteomic analysis

The trichloroacetic acid/acetone method was used for proteins extraction following Wu et al.^118^. The protein pellet was resuspended in 2 mL urea buffer (6 M urea and 4 mM calcium chloride in 200 mM 3-(*N*-morpholino) propanesulfonic acid (MOPS), pH 8.0)^120^. An equivalent amount of protein (100 μg) was reduced using 10 mM dithiothreitol (DTT) and alkylated in 40 mM iodoacetamide (IAA) in the dark. After alkylation, the concentration of urea in the mixture was reduced to less than 2 M by diluting with 4 mM CaCl_2_. The protein was digested with trypsin (1:20) followed by incubation at 37 °C overnight. Subsequently, the peptides were desalted utilising C18 SepPak reverse-phase cartridges and SWATH-MS analysis was performed^121^. The data was analysed from five biological repeats.

### Statistical analysis

Significant differences in data between different samples were analyzed by the Student’s *t*-test.

## Supplementary information

Supplementary file 1
