# Supplementary material for: The overexpression of OsACBP5 protects transgenic rice against necrotrophic, hemibiotrophic and biotrophic pathogens
Source: Sci Rep. 2020 Sep 10;10:14918. doi: 10.1038/s41598-020-71851-9 (PMC7483469; doi:10.1038/s41598-020-71851-9)
Supplement: Supplementary file 1 [file 41598_2020_71851_MOESM1_ESM.docx]

Original Research Article

**The overexpression of OsACBP5 protects transgenic rice against necrotrophic, hemibiotrophic and biotrophic pathogens**

**Saritha Panthapulakkal Narayanan, Shiu-Cheung Lung, Pan Liao, Clive Lo and Mee-Len Chye^*^**

School of Biological Sciences, The University of Hong Kong, Pokfulam Road, Hong Kong, China.

**SUPPORTING INFORMATION**

**Generation of OsACBP5**-**overexpressing (OE) transgenic rice**

The 1.7-kb *Xba*I-*Bam*HI full-length cDNA encoding *Oryza sativa* ACYL-COA-BINDING PROTEIN5 (OsACBP5) from plasmid pOS581^83^ was cloned into the *Xba*I-*Bam*HI site of binary vector pCAMBIA1304 to generate pOS879 of which *OsACBP5* is driven by the Cauliflower Mosaic Virus *35S* promoter. The pOS879 was sent to BioRun (http://www.biorun.net) for *Agrobacterium*-mediated rice transformation to generate OsACBP5-OE transgenic rice. T_3_-generation seeds from OsACBP5-OE transgenic rice were used for further experiments.

**Generation of OsACBP5-OE9*osnpr1* and OsACBP5-OE9*oscoi1* rice plants**

OsACBP5-OE9 was used as the pollen recipient in a cross with pollen donor *osnpr1* (T-DNA insertion mutant) to generate OsACBP5-OE9*osnpr1* transgenic rice plants. Similarly, OsACBP5-OE9 was crossed with the T-DNA insertion mutant, *oscoi1* to produce OsACBP5-OE9*oscoi1* transgenic rice plants. Both progeny lines were confirmed by PCR genotyping. T_3_-generation seeds from OsACBP5-OE9*osnpr1* and OsACBP5-OE9*oscoi1* transgenic rice were used for further experiments.

**Construction of the *OsACBP5pro::GUS* fusion and its deletion derivatives**

A 2.2-kb 5'-flanking region of *OsACBP5* (LOC_Os03g14000, RiceGE database <http://signal.salk.edu>) and its two 5'-truncated derivatives were fused to the *GUS* (encoding β-glucuronidase) reporter gene. Polymerase chain reactions (PCR) was performed using rice genomic DNA as a template to amplify the *OsACBP5* 5'-flanking fragments with various primer pairs (Supplemental Table S2). All forward primers contain a *Hind*III restriction site and the reverse primer a *Bam*HI restriction site. Fragments were purified and cloned into a pGEM-T-Easy vector (Promega). Each *Hind*III-*Bam*HI fragment was subcloned into corresponding restriction sites on the binary vector DX2181 to generate three *OsACBP5pro::GUS* fusions. The resultant plasmids designated as pOS820, pOS891 and pOS895, respectively, were sent to BioRun (http://www.biorun.net) for *Agrobacterium*-mediated transformation to generate *OsACBP5pro::GUS* transgenic rice. T_3_-generation seeds from pOS820, pOS891 and pOS895 transformants were used for further experiments.

**Disease assays**

*Rhizoctonia solani* AG-1-1 (ATCC 66157) was cultured onto potato-dextrose agar (PDA) plates for incubation at 28^o^C for 7 days. Five-week-old rice plants were inoculated by placing a fungal mycelial ball beneath the leaf sheath. The inoculated plants were covered with a plastic bag for 2 weeks^57^. Disease assessment was scored by measuring lesion length^57^.

*Cercospora oryzae* (ATCC 60687) was grown in PDA for 2 weeks at 25^o^C. Three-week-old rice plants were inoculated by spraying (10^5^ spores mL^-1^)^131^. Disease severity was rated using a 0-9 scale, where 0 = no disease, 1 = 1%, 2 = 3%, 3 = 5%, 4 = 12%, 5 = 25%, 6 = 40%, 7 = 65%, 8 = 75% and 9 = more than 75% diseased leaf area^132^.

*Magnaporthe grisea* (RB22) was cultured onto oat meal agar for 2 weeks in the dark at 28 to 30°C and then exposed to fluorescent light for 1 week at RT for sporulation^132^. Three-week-old rice plants were spray inoculated with 5 X 10^5^ spores mL^-1^ with 0.02% Tween-20^132^. Disease severity was analyzed by measuring the lesion area in the infected leaves.

*Fusarium graminearum* (ATCC MYA-4620) was cultured on potato dextrose agar (PDA) plates at 25°C for 7 days^133^. The seeds were surface-sterilized and imbibed in *F. graminearum* spore suspension (4×10^4^ spores mL^-1^) for 15 min and transferred onto Petri plates with moist sterile filter paper and stored at 15°C^134^. After overnight incubation, the seeds were planted in soil in the greenhouse. The number of seeds germinated was recorded as seedlings with coleoptiles length more than 1 cm at 7 dpi^134^.

*Xanthomonas oryzae* (*Xoo*) (PXO99A) was grown in peptone sucrose agar and incubated at 28°C for three days. At the day of inoculation, the *Xoo* bacterial mass was suspended in 10 mM magnesium chloride (MgCl_2_) at a concentration of OD_600_ = 0.5. Three-week-old rice plants were inoculated with *Xoo* suspension using the leaf-clipping method^135^. Leaf length was measured 14 dpi.

**Phytohormone treatments**

Three-week-old rice seedlings of the T_3_-generation derived from transformation using *OsACBP5pro::GUS* fusion constructs pOS820, pOS891 and pOS895 were submerged in 100 µM salicylic acid (SA) (Sigma-Aldrich)/ methyl jasmonate (MeJA) (Sigma-Aldrich) according to Nakashima *et al.*^136^. Samples were collected at different time intervals (0 h, 5 h, 12 h and 24 h) for fluorometric assays of GUS activity.

**Fluorometric assays of GUS activity**

Plant samples harvested from three-week-old pOS820, pOS891 and pOS895 transformants (T_3_-generation) were homogenized in liquid nitrogen and resuspended in 500 μl GUS extraction buffer (50 mM sodium phosphate pH 7.0, 10 mM ethylenediaminetetraacetic acid (EDTA) pH 8.0, 0.1 % SDS, 0.1 % Triton X-100) following Jefferson *et al.*^137^. The supernatant after centrifugation was transported to a fresh Eppendorf tube and the Bradford protein assay^138^ was performed to determine protein concentration. The supernatant (50 μl) was mixed with preheated (37 °C) reaction mix solution (250 μl) containing 2 mM 4-methylumbelliferyl D-glucuronide (4-MUG) in GUS extraction buffer. A 50-μl aliquot from the reaction tube was added at 10-min intervals to a new Eppendorf new containing 950 μl stop reagent. Standards for quantification were prepared using 100 nM, 250 nM and 500 nM 4-methylumbelliferone (4-MU). GUS activity was examined with excitation wavelength of 365 nm and emission wavelength of 455 nm using a spectrofluorometer (Bio-Tek FL600, Bio-tek Instruments, Inc. USA). GUS activity was expressed as pmol 4-MU min^-1^ mg^-1^ protein^1^^37^.

**Electrophoretic mobility shift assays (EMSAs)**

Twenty-bp DNA probes were labelled with the Biotin 3′-End DNA Labelling Kit (Thermo). Labelling efficiency was estimated before EMSA studies. LightShift Chemiluminescent EMSA Kit (Thermo, Rockford, IL, USA) was used to perform EMSAs. Plant nuclear proteins were extracted from WT *O. sativa* cv Zhonghua11 (ZH11) seeds and leaves using the NE-PER Nuclear and Cytoplasmic Extraction Kit (Thermo Scientific). Nuclear proteins and the DNA probe were incubated in binding buffer for 20 minutes at RT using the Thermo Scientific Pierce Biotin 3'-End DNA Labelling Kit. An unlabelled DNA probe was used as a competitor. Samples were separated on 6% native polyacrylamide gel and transferred to a Hybond-N membrane. The blot was detected using LightShift Chemiluminescent EMSA Kit following the manufacturer’s instruction.

**Isothermal titration calorimetry (ITC) experiments**

To study the binding of (His)_6_-OsACBP5 to 18:3-acyl-CoA ester, ITC was performed with a MicroCal iTC200 system (GE Healthcare) at 30^o^C. The 18:3-acyl-CoA ester (Avanti Polar Lipids Inc.) was dissolved in 10 mM sodium phosphate buffer (pH 7.0) to a final concentration of 300 mM. The solution was then loaded into a syringe with the sample cell containing 30 mM OsACBP5. Titration was carried out through 20 successive 1.8 µl injections over 4 s at 150 s intervals with a stirring speed of 1000 rev min^-1^. Sodium phosphate buffer (10 mM) was used as a negative control. Data processing was carried out by estimating nonspecific heat effects after saturation following the instructions of the manufacturer. Raw data were integrated and analyzed using the Origin v.7.0 according to the directions in the software manual.

**Quantitative real-time polymerase chain reactions (qRT-PCR)**

Total RNA was extracted from rice seedlings using the RNeasy Plant Mini Kit (Qiagen). DNase I (Qiagen) was used to treat the RNA (5 μg) before reverse-transcription into first-strand cDNA using the SuperScript First-Strand Synthesis System (Invitrogen). qRT-PCR was carried out with a StepOne Plus Real-time PCR System (Applied Biosystems) and FastStart Universal SYBR Green Mater (Roche). The conditions for qRT-PCR were as follows: denaturation at 95^o^C for 10 min, followed by 40 cycles of 95^o^C for 15 s and 60^o^C for 1 min. Three experimental replicates for each reaction were performed using gene-specific primers. The internal control used was rice *ACTIN*. The qRT-PCR data were analysed by the comparative C_T_ method^139^. The primers for qRT-PCR are listed in Supplemental Table S2.


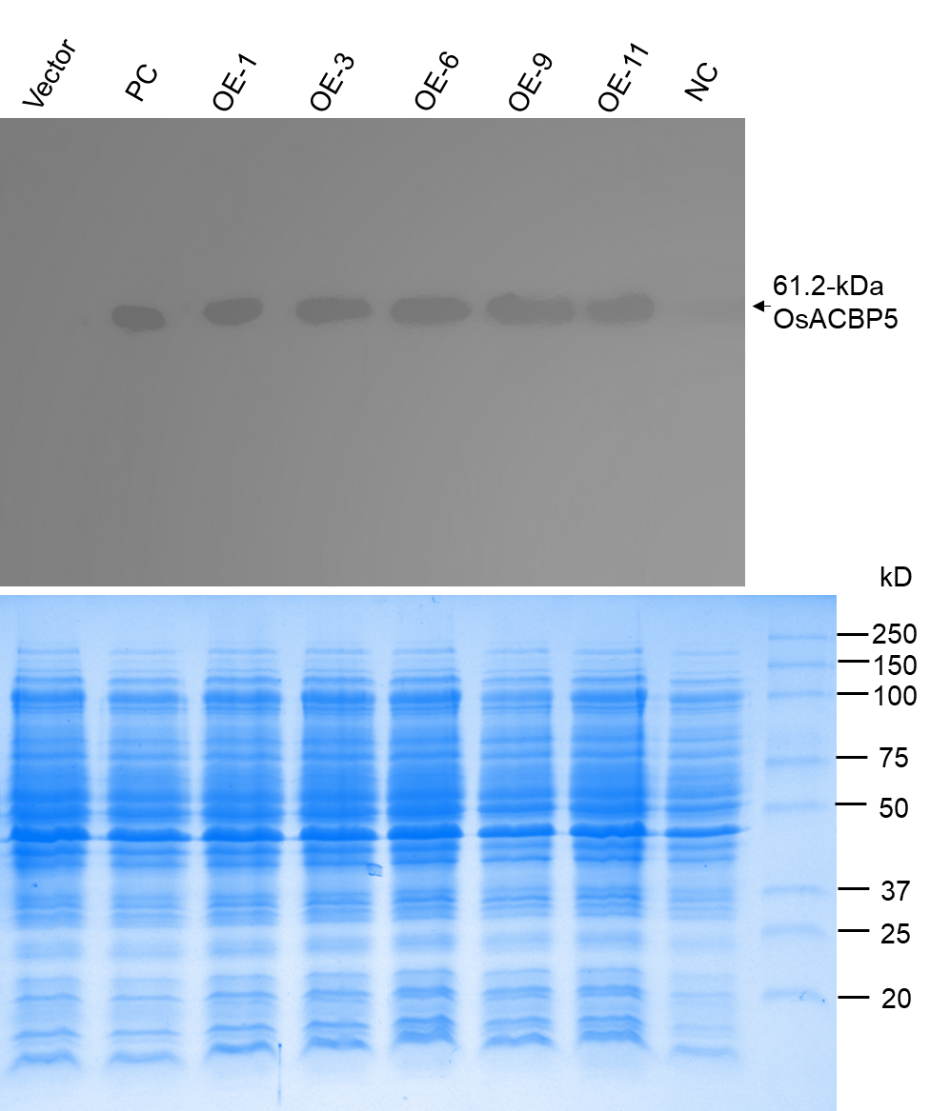


**Figure S1.** Western blot analysis using antibodies against OsACBP5 to verify the expression of OsACBP5 in 3-week-old transgenic rice OsACBP5-OE leaves. The cross-reacting OsACBP5 band is indicated by an arrow. OsACBP5-OEs were designated as OE-1, OE-3, OE-6, OE-9 and OE-11; PC, positive control (transgenic Arabidopsis OsACBP5-OE as reported in Meng and Chye, 2014); NC, negative control (transgenic Arabidopsis AtACBP3-OE) vector, vector (pCAMBIA1304)-transformed rice. Bottom, Coomassie Blue-stained gel of 20 µg total protein in each well.


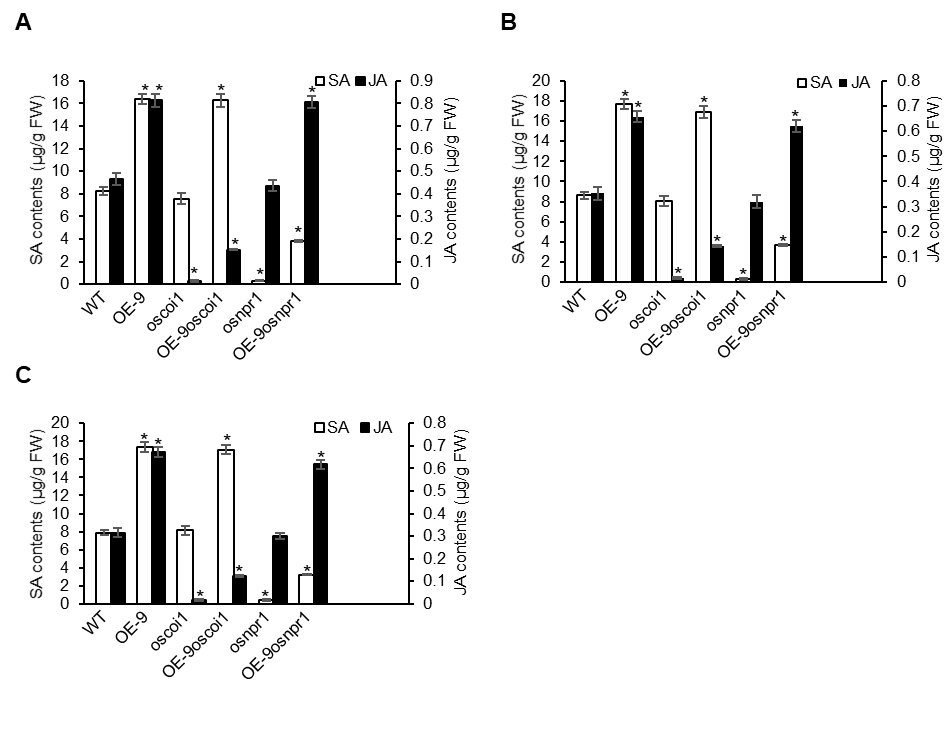


**Figure S2.** Salicylic acid (SA) and jasmonate (JA) contents in *Rhizoctonia solani-, Magnaporthe grisea-* and *Xoo-*infected wild-type (WT), transgenic rice overexpressing OsACBP5 (OE-9)*, osnpr1, oscoi1*, OE-9*osnpr1* and OE-9*oscoi1* plants.

(A) *R. solani-*infected *oscoi1* and OE-9*oscoi1* respectively showed 40- and three-fold lower JA content compared to the WT.

(B) *M. grisea-* and (C) *Xoo-*infected *osnpr1* and OE-9*osnpr1* respectively showed ~ 20- and 2.5-fold lower SA content compared to the WT.

Data points represent means ± SD from three independent experiments. FW, Fresh weight. Asterisks indicate significant difference (*P*<0.05) in comparison to the controls by Student’s *t-*test.


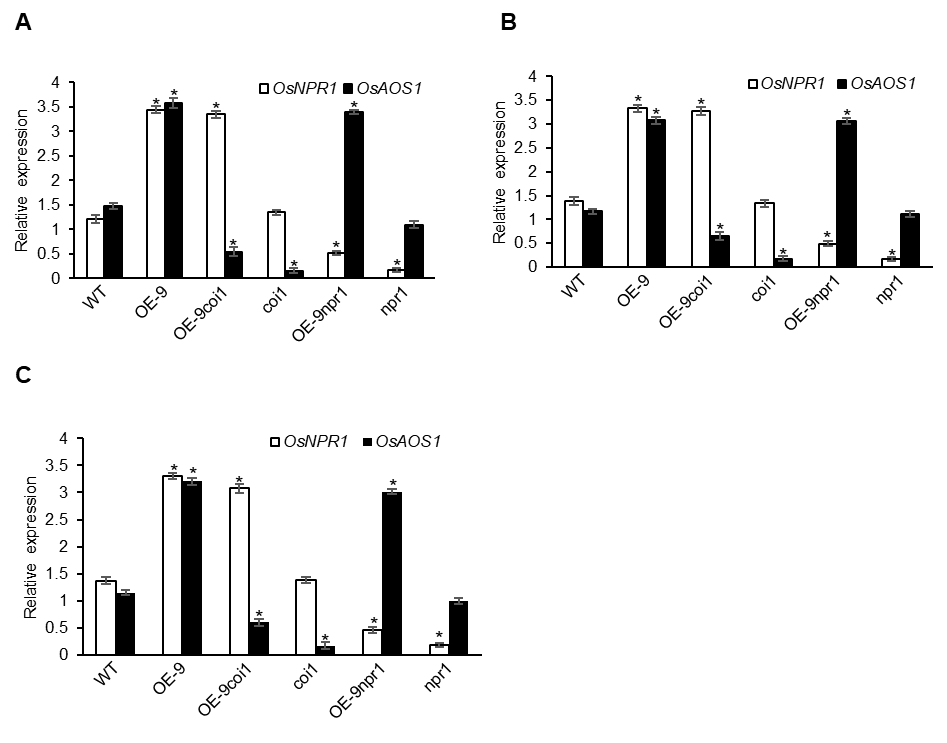


**Figure S3.** qRT-PCR analyses on the expression of *OsNPR1* and *OsAOS1* in

(A) *Rhizoctonia solani-*,

(B) *Magnaporthe grisea-* and,

(C) *Xoo-*infected

wild-type (WT), transgenic rice overexpressing OsACBP5 (OE-9)*, osnpr1, oscoi1*, OE-9*osnpr1* and OE-9*oscoi1* plants.


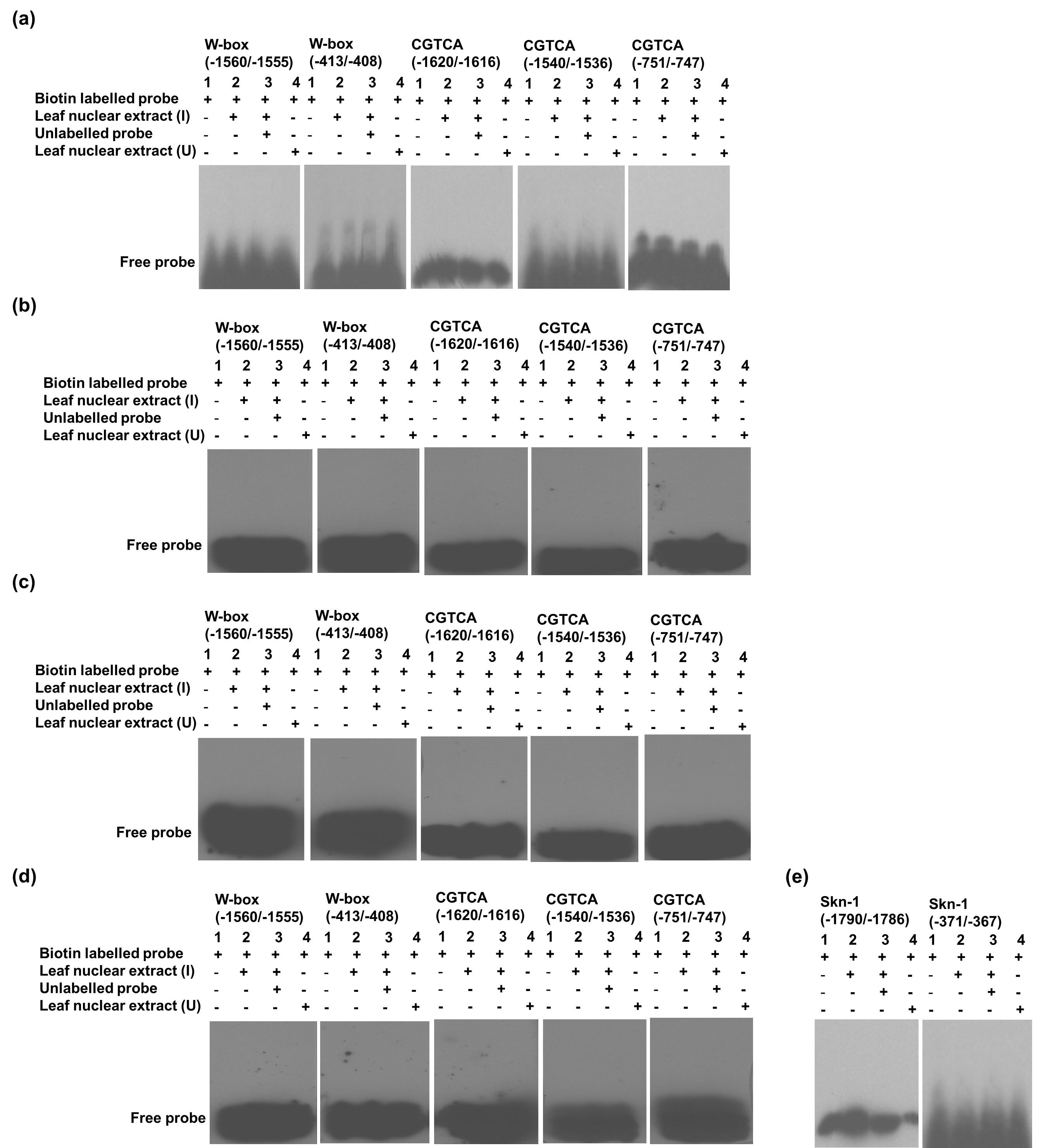


**Figure S4.** EMSAs on W-boxes (-1560/-1555, -413/-408), CGTCA (-1620/-1616, -1540/-1536) and Skn-1 boxes (-1790/-1786, -371/-367).

(a) Interaction of leaf nuclear extracts from *R. solani*-infected five-week-old wild-type (WT) rice leaves with W-box (-1560/-1555, -413 /-408) and CGTCA (-1620/-1616, -1540/-1536 and -751/-747) probes.

(b) Interaction of leaf nuclear extracts from *M. grisea-*infected three-week-old WT rice leaves with W-box (-1560/-1555, -413 /-408) and CGTCA (-1620/-1616, -1540/-1536 and -751/-747) probes.

(c) Interaction of leaf nuclear extracts from *Xoo*-infected three-week-old WT rice leaves with W-box (-1560/-1555, -413 /-408) and CGTCA (-1620/-1616, -1540/-1536 and -751/-747) probes.

(d) Interaction of leaf nuclear extracts from *C. oryzae*-infected three-week-old WT rice leaves with W-box (-1560/-1555, -413 /-408) and CGTCA (-1620/-1616, -1540/-1536 and -751/-747) probes.

(e) Interaction of seed nuclear extracts from *F. oxysporum*-infected WT rice seeds with Skn-1 motif (-1790/-1786 and -371/-367) probes.

Lane 1, free probe without the addition of crude nuclear extracts. Crude nuclear extracts from infected (I) leaves were incubated with biotin end-labelled probes (lane 2) in the presence of a 500-fold molar excess of unlabelled competitor (lane 3). Lane 4, a negative control with uninfected (U) leaf nuclear extract.


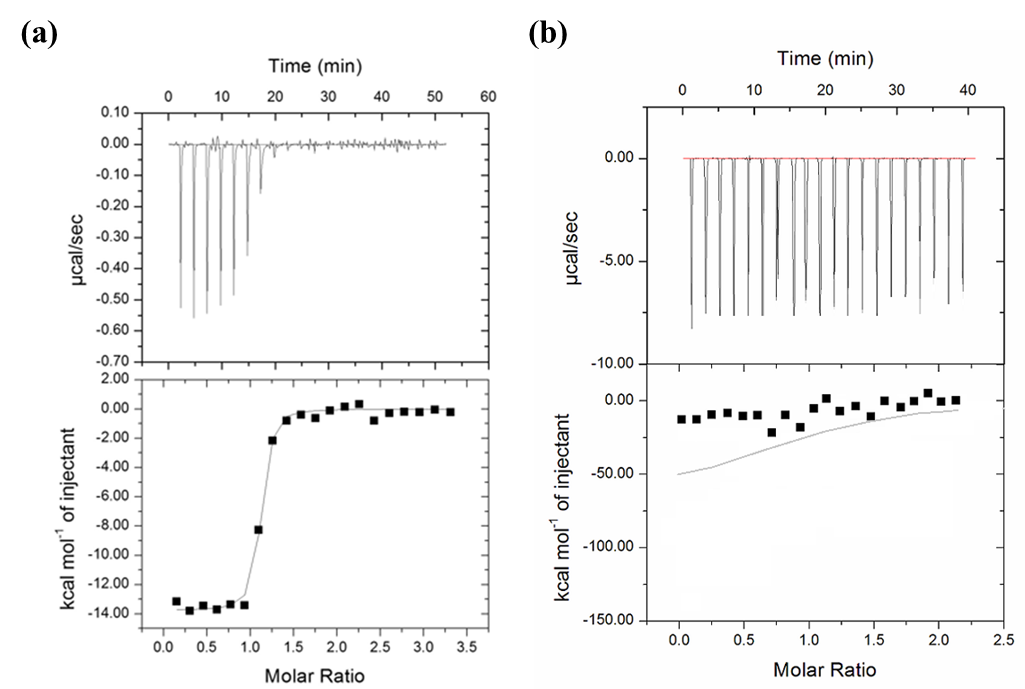


**Figure S5.** Binding isotherm of recombinant OsACBP5 titrated with 18:3-CoA ester at 30 °C. Raw data of 300 μl of 30 mM recombinant OsACBP5 titrated with 20 injections of 1.5 μl of 300 mM **(a)**18:3-CoA ester and **(b)** sodium phosphate buffer (10 mM; negative control) is represented in the top panel. The bottom panel shows the integrated area of each injection after background correction.


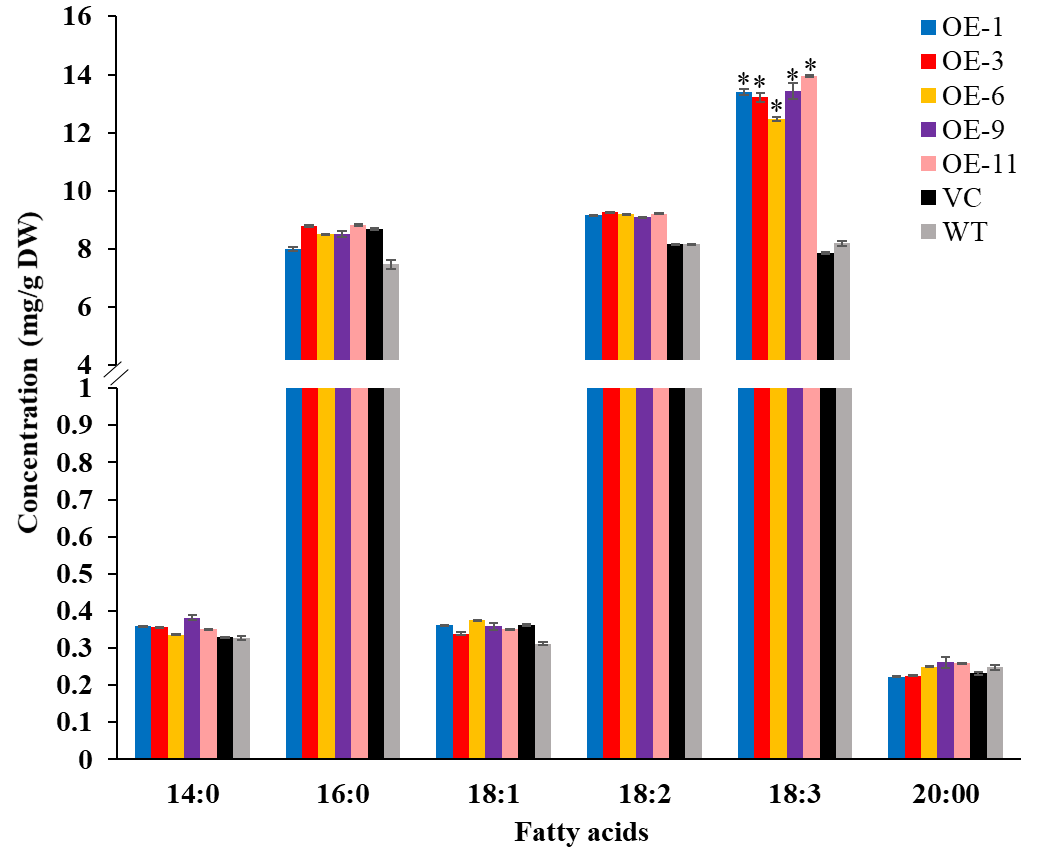


**Figure S6.** Gas chromatography-mass spectrometry analysis of fatty acids from OsACBP5-OE transgenic rice leaves.

Quantitative analysis of fatty acids (14:0, 16:0, 18:1, 18:2, 18:3 and 20:0-FA) from OsACBP5-OEs (OE-1 (blue), OE-3 (red), OE-6 (orange), OE-9 (violet), OE-11 (rose)), pCAMBIA1304 vector-transformed control (black) and wild type (grey). Data points represent means ± SD from three independent experiments. DW, Dry weight. Asterisks indicate significant difference (*P*<0.05) in comparison to the controls by the Student's *t-*test.


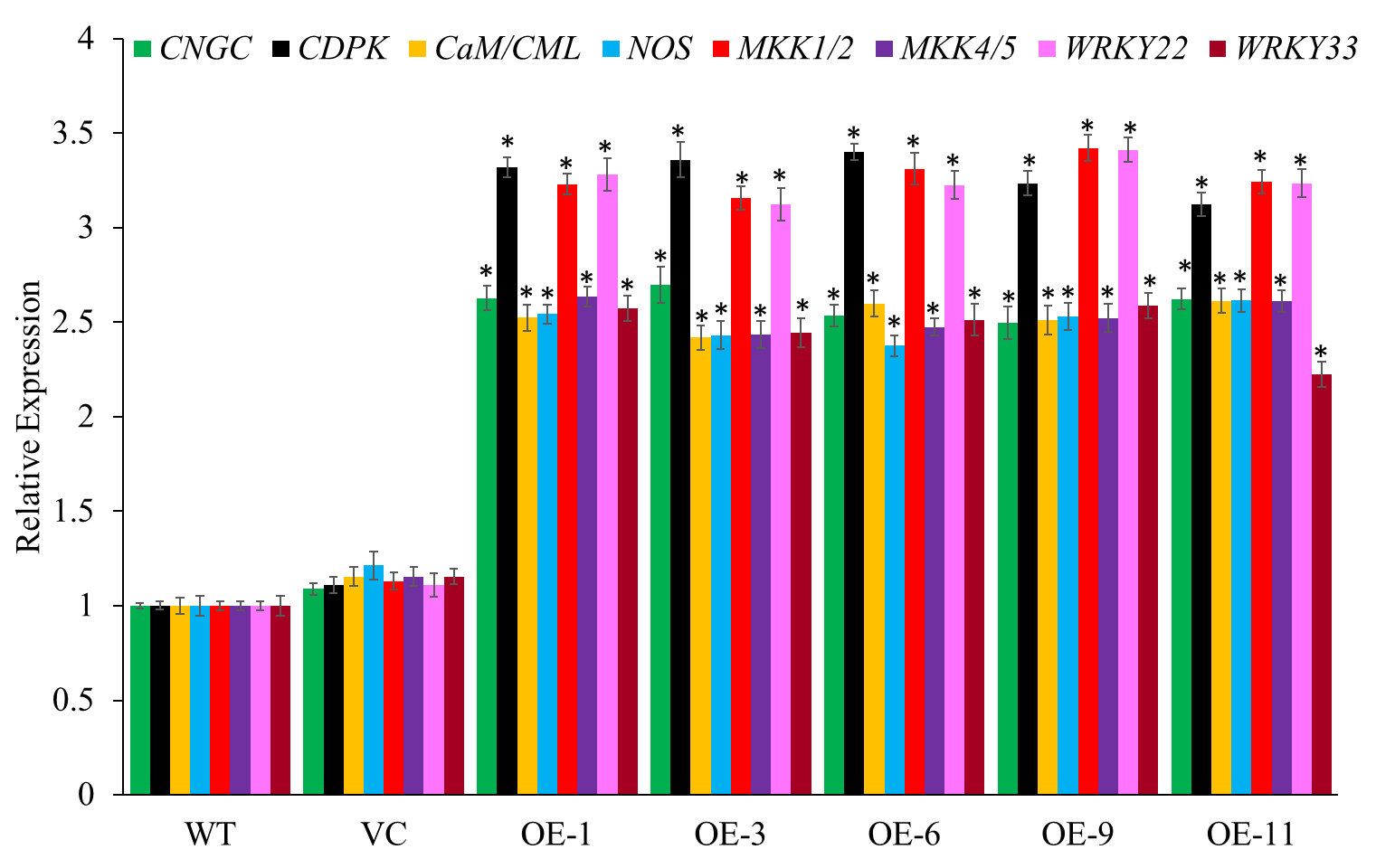


**Figure S7.** qRT-PCR analyses of genes related to PTI in OsACBP5-OEs in comparison to the wild type (WT) and vector-transformed control (VC).

The genes analysed were *CNGC, CDPK, CaM/CML, NOS, MKK1/2, MKK4/5, WRKY22* and *WRKY33*. The bar graph shows relative gene expression in *R. solani-*infected rice OsACBP5-OEs (OE-1, OE-3, OE-6, OE-9 and OE-11) compared to the WT and VC. The expression levels were normalized to that of *ACTIN*. Data points represent means ± SD from three independent experiments. Asterisks indicate significant difference (*P*<0.05) in comparison to the controls by Student's *t-*test. *CaM/CML, CALMODULIN/CALMODULIN-LIKE PROTEINS; CDPK, CALCIUM-DEPENDENT PROTEIN KINASE; CNGC, CYCLIC NUCLEOTIDE GATED CHANNELS; MKK1/2, MITOGEN-ACTIVATED PROTEIN KINASE KINASE1/2; MKK4/5, MITOGEN-ACTIVATED PROTEIN KINASE KINASE4/5; NOS, NITRIC OXIDE SYNTHASE; WRKY 22, WRKY TRANSCRIPTION FACTOR22; WRKY33, WRKY TRANSCRIPTION FACTOR33.*


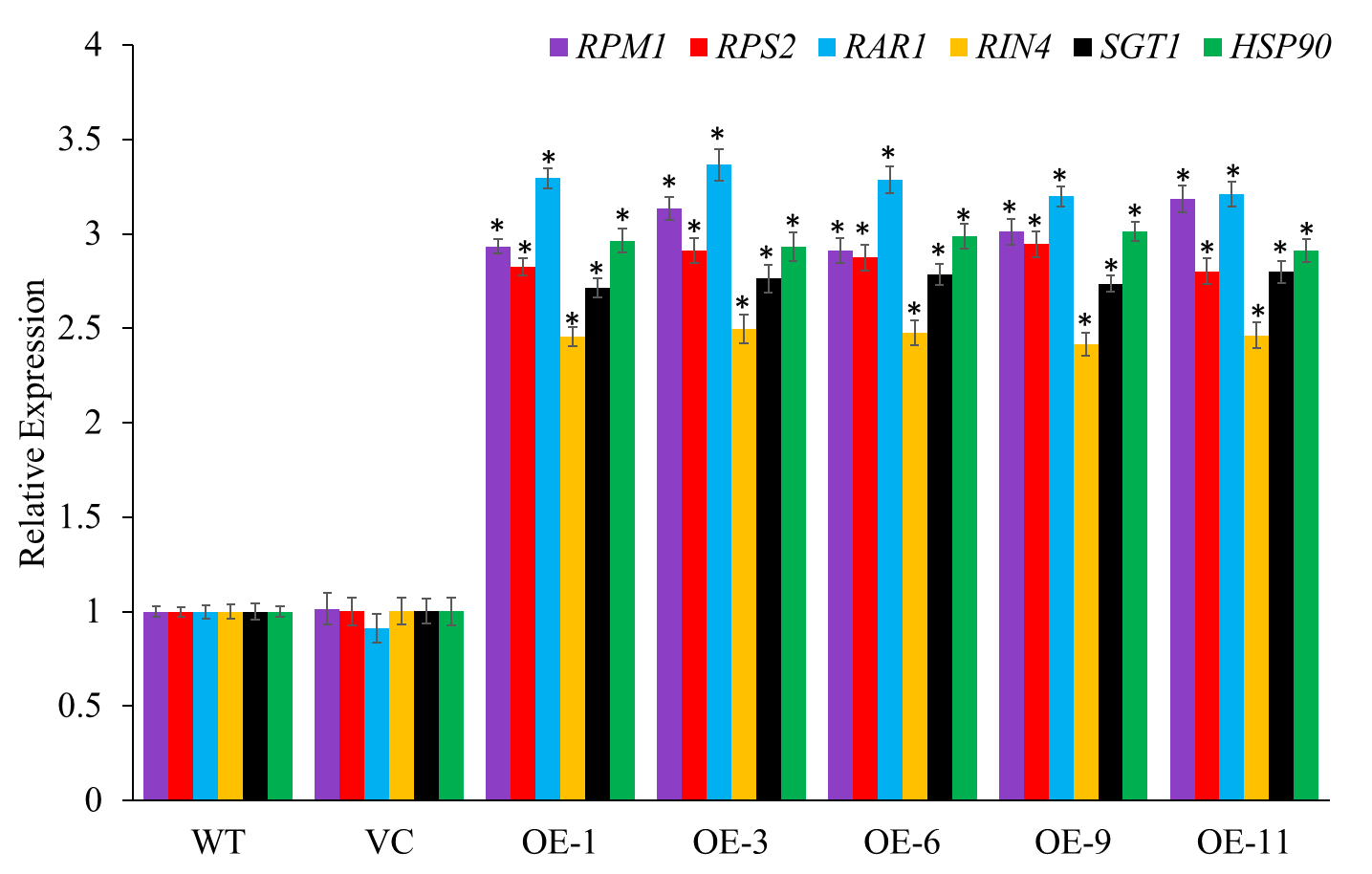


**Figure S8.** qRT-PCR analyses of genes related to ETI in OsACBP5-OEs in comparison to the wild type (WT) and vector-transformed control (VC).

The genes analysed were *RPM1, RPS2, RAR1, RIN4, SGT1* and *HSP90*. The bar graph shows relative gene expression in *R. solani-*infected rice OsACBP5-OEs (OE-1, OE-3, OE-6, OE-9 and OE-11) compared to the WT and VC. The expression levels were normalized to that of *ACTIN*. Data points represent means ± SD from three independent experiments. Asterisks indicate significant difference (*P*<0.05) in comparison to the controls by Student's *t-*test. *HSP90, HEAT SHOCK PROTEIN90; RAR1, RPM1, RPS2, RPS5, DISEASE RESISTANT PROTEINS; RIN4, RPM1-INTERACTING PROTEIN4; SGT1, SUPPRESSOR OF G2 ALLELE OF SKP1*.


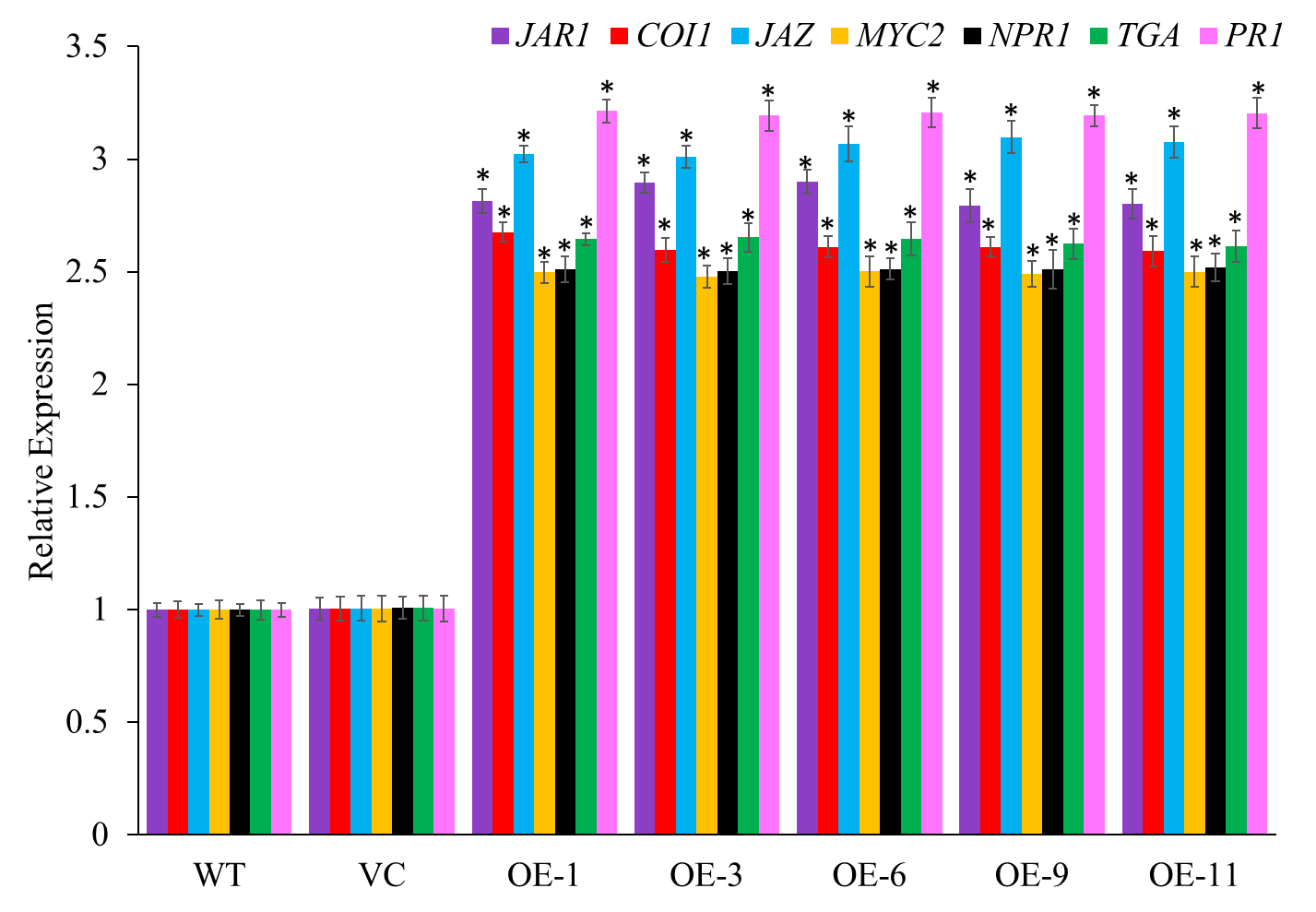


**Figure S9.** qRT-PCR analyses of genes associated with the SA- and JA-signalling pathways in OsACBP5-OEs in comparison to the wild type (WT) and vector-transformed control (VC).

The genes analysed were *JAR1, COI1, JAZ* and MYC2 (associated with JA-signalling pathway) and *NPR1, TGA* and *PR1* (associated with SA-signalling pathway). The bar graph shows relative gene expression in *R. solani-*infected rice OsACBP5-OEs (OE-1, OE-3, OE-6, OE-9 and OE-11) compared to the WT and VC. The expression levels were normalized to that of *ACTIN*. Data points represent means ± SD from three independent experiments. Asterisks indicate significant difference (*P*<0.05) in comparison to the controls by Student's *t-*test. *COI1, CORONATINE INSENSITIVE PROTEIN1; JAR1, JASMONOYL ISOLEUCINE CONJUGATE SYNTHASE1; JAZ, JASMONATE ZIM-DOMAIN CONTAINING PROTEIN; MYC2, TRANSCRIPTION FACTOR MYC2, NPR1, NON-EXPRESSOR OF PATHOGENESIS-RELATED1; PR1, PATHOGENESIS-RELATED PROTEIN1; TGA, TRANSCRIPTION FACTOR TGA.*

**Figure S10.** Expression of *ACTIN* in *R. solani-*infected wild type (WT), vector-transformed control (VC) and OsACBP5-OEs (OE-1, OE-3, OE-6, OE-9, OE-11). The expression of *ACTIN* in WT, VC and OsACBP5-OEs was calculated from mean C_T_ values. Data points represent means ± SD from three biological replicates.

**Table S1.** Thermodynamic parameters for 18:3-CoA binding to recombinant OsACBP5. The values are the means ± SD from three independent experiments. n, stoichiometry of the ligand-to-protein binding; *ΔH,* enthalpy change; *ΔS,* entropy change; *ΔG,* free energy change; *K_d_,* dissociation constant.

| *n* | *ΔH* (kcal mol^-1^) | *ΔS* (cal mol^-1^) | *ΔG* (kcal mol^-1^) | *K_d_* (nM) |
| --- | --- | --- | --- | --- |
| 1.01 ± 0.03 | -10.68 ± 0.55 | -2.45 | -9.94 ± 0.22 | 59.5 ± 5.1 |

**Table S2.** Differentially expressed genes (DEGs) associated with the plant-pathogen interaction pathway as well as SA- and JA-signalling pathways in *R. solani*-infected transgenic rice OsACBP5-OEs. Genes with fold change >1.0 are considered upregulated.

| **Locus** | **Gene** | **Fold change** | ***p-*value** |
| --- | --- | --- | --- |
| LOC_Os12g28270 | *CYCLIC NUCLEOTIDE GATED CHANNELS* | 1.04 | 0.003 |
| LOC_Os03g55100 | *CYCLIC NUCLEOTIDE GATED CHANNELS* | 1.09 | 0.013 |
| LOC_Os02g54760 | *CYCLIC NUCLEOTIDE GATED CHANNELS* | 2.15 | 0.016 |
| LOC_Os09g38580 | *CYCLIC NUCLEOTIDE GATED CHANNELS* | 3.77 | 0.008 |
| LOC_Os04g58860 | *CALCIUM-DEPENDENT PROTEIN KINASE* | 3.55 | 0.005 |
| LOC_Os07g22710 | *CALCIUM-DEPENDENT PROTEIN KINASE* | 3.37 | 0.003 |
| LOC_Os05g05460 | *CALMODULIN/CALMODULIN-LIKE PROTEINS* | 1.74 | 0.018 |
| LOC_Os11g04480 | *CALMODULIN/CALMODULIN-LIKE PROTEINS* | 1.64 | 0.004 |
| LOC_Os06g40720 | *CALMODULIN/CALMODULIN-LIKE PROTEINS* | 1.07 | 0.011 |
| LOC_Os10g25010 | *CALMODULIN/CALMODULIN-LIKE PROTEINS* | 1.96 | 0.006 |
| LOC_Os01g72080 | *CALMODULIN/CALMODULIN-LIKE PROTEINS* | 1.81 | 0.007 |
| LOC_Os12g04240 | *CALMODULIN/CALMODULIN-LIKE PROTEINS* | 1.08 | 0.014 |
| LOC_Os03g21380 | *CALMODULIN/CALMODULIN-LIKE PROTEINS* | 1.64 | 0.007 |
| LOC_Os09g28480 | *CALMODULIN/CALMODULIN-LIKE PROTEINS* | 1.09 | 0.004 |
| LOC_Os08g02420 | *CALMODULIN/CALMODULIN-LIKE PROTEINS* | 1.49 | 0.012 |
| LOC_Os02g10470 | *CALMODULIN/CALMODULIN-LIKE PROTEINS* | 1.06 | 0.017 |
| LOC_Os04g39320 | *CALMODULIN/CALMODULIN-LIKE PROTEINS* | 1.10 | 0.004 |
| LOC_Os12g04360 | *CALMODULIN/CALMODULIN-LIKE PROTEINS* | 2.05 | 0.002 |
| LOC_Os11g04560 | *CALMODULIN/CALMODULIN-LIKE PROTEINS* | 1.06 | 0.007 |
| LOC_Os05g31620 | *CALMODULIN/CALMODULIN-LIKE PROTEINS* | 1.07 | 0.016 |
| LOC_Os05g50180 | *CALMODULIN/CALMODULIN-LIKE PROTEINS* | 2.01 | 0.019 |
| LOC_Os08g44390 | *CALMODULIN/CALMODULIN-LIKE PROTEINS* | 2.38 | 0.003 |
| LOC_Os08g35210 | *RESPIRATORY BURST OXIDASE HOMOLOG* | 2.30 | 0.012 |
| LOC_Os09g26660 | *RESPIRATORY BURST OXIDASE HOMOLOG* | 2.15 | 0.005 |
| LOC_Os11g33120 | *RESPIRATORY BURST OXIDASE HOMOLOG* | 1.21 | 0.004 |
| LOC_Os01g53294 | *RESPIRATORY BURST OXIDASE HOMOLOG* | 1.11 | 0.007 |
| LOC_Os06g38970 | *FLAGELLIN-SENSING2* | 1.80 | 0.016 |
| LOC_Os03g58110 | *FLAGELLIN-SENSING2* | 2.05 | 0.006 |
| LOC_Os08g10320 | *FLAGELLIN-SENSING2* | 1.02 | 0.017 |
| LOC_Os03g11340 | *FLAGELLIN-SENSING2* | 1.48 | 0.014 |
| LOC_Os06g28480 | *FLAGELLIN-SENSING2* | 1.08 | 0.005 |
| LOC_Os10g33130 | *FLAGELLIN-SENSING2* | 1.06 | 0.007 |
| LOC_Os11g40970 | *FLAGELLIN-SENSING2* | 1.04 | 0.007 |
| LOC_Os12g12010 | *FLAGELLIN-SENSING2* | 1.10 | 0.011 |
| LOC_Os11g47170 | *FLAGELLIN-SENSING2* | 1.86 | 0.004 |
| LOC_Os08g24310 | *FLAGELLIN-SENSING2* | 1.16 | 0.007 |
| LOC_Os02g12010 | *FLAGELLIN-SENSING2* | 1.07 | 0.008 |
| LOC_Os07g07990 | *FLAGELLIN-SENSING2* | 2.22 | 0.012 |
| LOC_Os02g54600 | *MITOGEN-ACTIVATED PROTEIN KINASE KINASE* | 2.17 | 0.005 |
| LOC_Os02g41460 | *MITOGEN-ACTIVATED PROTEIN KINASE KINASE* | 1.92 | 0.010 |
| LOC_Os02g53040 | *MITOGEN-ACTIVATED PROTEIN KINASE KINASE* | 1.23 | 0.007 |
| LOC_Os01g32660 | *MITOGEN-ACTIVATED PROTEIN KINASE KINASE* | 1.08 | 0.006 |
| LOC_Os06g09180 | *MITOGEN-ACTIVATED PROTEIN KINASE KINASE* | 1.10 | 0.017 |
| LOC_Os10g33780 | *MITOGEN-ACTIVATED PROTEIN KINASE KINASE* | 1.63 | 0.009 |
| LOC_Os05g28040 | *MITOGEN-ACTIVATED PROTEIN KINASE KINASE* | 1.95 | 0.004 |
| LOC_Os02g08440 | *WRKY TRANSCRIPTION FACTOR33* | 2.54 | 0.002 |
| LOC_Os01g18584 | *WRKY TRANSCRIPTION FACTOR33* | 1.01 | 0.015 |
| LOC_Os12g32250 | *WRKY TRANSCRIPTION FACTOR33* | 1.05 | 0.017 |
| LOC_Os05g27730 | *WRKY TRANSCRIPTION FACTOR33* | 1.29 | 0.008 |
| LOC_Os11g29870 | *WRKY TRANSCRIPTION FACTOR33* | 2.82 | 0.005 |
| LOC_Os01g14440 | *WRKY TRANSCRIPTION FACTOR33* | 2.06 | 0.019 |
| LOC_Os05g09020 | *WRKY TRANSCRIPTION FACTOR33* | 1.05 | 0.013 |
| LOC_Os06g44010 | *WRKY TRANSCRIPTION FACTOR33* | 1.61 | 0.005 |
| LOC_Os05g46020 | *WRKY TRANSCRIPTION FACTOR33* | 1.46 | 0.008 |
| LOC_Os01g47560 | *WRKY TRANSCRIPTION FACTOR33* | 2.13 | 0.003 |
| LOC_Os05g49100 | *WRKY TRANSCRIPTION FACTOR33* | 1.41 | 0.017 |
| LOC_Os01g09100 | *WRKY TRANSCRIPTION FACTOR33* | 2.74 | 0.004 |
| LOC_Os05g50610 | *WRKY TRANSCRIPTION FACTOR33* | 2.36 | 0.019 |
| LOC_Os01g09080 | *WRKY TRANSCRIPTION FACTOR33* | 2.68 | 0.014 |
| LOC_Os02g53100 | *WRKY TRANSCRIPTION FACTOR33* | 1.36 | 0.009 |
| LOC_Os01g43650 | *WRKY TRANSCRIPTION FACTOR33* | 2.69 | 0.006 |
| LOC_Os04g21950 | *WRKY TRANSCRIPTION FACTOR22* | 1.14 | 0.017 |
| LOC_Os01g54600 | *WRKY TRANSCRIPTION FACTOR22* | 1.50 | 0.018 |
| LOC_Os03g53050 | *WRKY TRANSCRIPTION FACTOR22* | 1.05 | 0.006 |
| LOC_Os05g03900 | *WRKY TRANSCRIPTION FACTOR22* | 1.73 | 0.005 |
| LOC_Os08g13840 | *WRKY TRANSCRIPTION FACTOR22* | 1.16 | 0.011 |
| LOC_Os01g53040 | *WRKY TRANSCRIPTION FACTOR22* | 2.07 | 0.017 |
| LOC_Os01g60640 | *WRKY TRANSCRIPTION FACTOR22* | 1.04 | 0.013 |
| LOC_Os03g63140 | *RPM1-INTERACTING PROTEIN4* | 1.13 | 0.012 |
| LOC_Os09g07920 | *RPM1-INTERACTING PROTEIN4* | 1.01 | 0.006 |
| LOC_Os06g17870 | *RPM1-INTERACTING PROTEIN4* | 1.05 | 0.015 |
| LOC_Os08g41470 | *RPM1-INTERACTING PROTEIN4* | 1.50 | 0.016 |
| LOC_Os11g29210 | *RPM1-INTERACTING PROTEIN4* | 2.51 | 0.018 |
| LOC_Os11g11990 | *DISEASE RESISTANT PROTEIN RPM1* | 1.09 | 0.007 |
| LOC_Os03g20550 | *DISEASE RESISTANT PROTEIN RPM1* | 3.76 | 0.008 |
| LOC_Os11g12050 | *DISEASE RESISTANT PROTEIN RPM1* | 1.53 | 0.014 |
| LOC_Os12g02420 | *DISEASE RESISTANT PROTEIN RPM1* | 1.02 | 0.010 |
| LOC_Os11g02480 | *DISEASE RESISTANT PROTEIN RPM1* | 1.14 | 0.019 |
| LOC_Os04g39350 | *DISEASE RESISTANT PROTEIN RPS2* | 1.88 | 0.006 |
| LOC_Os05g49620 | *DISEASE RESISTANT PROTEIN RPS2* | 1.01 | 0.008 |
| LOC_Os04g43440 | *DISEASE RESISTANT PROTEIN RPS2* | 4.07 | 0.015 |
| LOC_Os04g39320 | *DISEASE RESISTANT PROTEIN RPS2* | 7.41 | 0.008 |
| LOC_Os03g20550 | *DISEASE RESISTANT PROTEIN RPS2* | 2.53 | 0.005 |
| LOC_Os02g33180 | *DISEASE RESISTANT PROTEIN RAR1* | 1.05 | 0.012 |
| LOC_Os06g50300 | *DISEASE RESISTANT PROTEIN HSP90* | 1.12 | 0.006 |
| LOC_Os09g29840 | *DISEASE RESISTANT PROTEIN HSP90* | 1.06 | 0.012 |
| LOC_Os01g43540 | *DISEASE RESISTANT PROTEIN SGT1* | 1.21 | 0.017 |
| LOC_Os05g23850 | *NON-EXPRESSOR OF PATHOGENESIS-RELATED1* | 1.04 | 0.004 |
| LOC_Os01g72020 | *NON-EXPRESSOR OF PATHOGENESIS-RELATED1* | 1.05 | 0.011 |
| LOC_Os01g56200 | *NON-EXPRESSOR OF PATHOGENESIS-RELATED1* | 5.83 | 0.008 |
| LOC_Os03g20310 | *TRANSCRIPTION FACTOR TGA* | 1.54 | 0.005 |
| LOC_Os07g48820 | *TRANSCRIPTION FACTOR TGA* | 1.90 | 0.006 |
| LOC_Os06g41100 | *TRANSCRIPTION FACTOR TGA* | 1.06 | 0.005 |
| LOC_Os08g07970 | *TRANSCRIPTION FACTOR TGA* | 1.60 | 0.002 |
| LOC_Os04g54474 | *TRANSCRIPTION FACTOR TGA* | 3.46 | 0.019 |
| LOC_Os07g03730 | *PATHOGENESIS-RELATED PROTEIN 1* | 5.25 | 0.006 |
| LOC_Os07g03590 | *PATHOGENESIS-RELATED PROTEIN 1* | 1.90 | 0.012 |
| LOC_Os07g14070 | *PATHOGENESIS-RELATED PROTEIN 1* | 1.07 | 0.006 |
| LOC_Os07g03750 | *PATHOGENESIS-RELATED PROTEIN 1* | 1.61 | 0.013 |
| LOC_Os07g03710 | *PATHOGENESIS-RELATED PROTEIN 1* | 5.55 | 0.017 |
| LOC_Os07g03368 | *PATHOGENESIS-RELATED PROTEIN 1* | 8.47 | 0.006 |
| LOC_Os07g03279 | *PATHOGENESIS-RELATED PROTEIN 1* | 7.33 | 0.008 |
| LOC_Os07g03600 | *PATHOGENESIS-RELATED PROTEIN 1* | 4.73 | 0.017 |
| LOC_Os05g50890 | *JASMONOYL ISOLEUCINE CONJUGATE SYNTHASE1* | 3.32 | 0.017 |
| LOC_Os05g37690 | *CORONATINE INSENSITIVE PROTEIN1* | 1.09 | 0.014 |
| LOC_Os03g15880 | *CORONATINE INSENSITIVE PROTEIN1* | 1.13 | 0.009 |
| LOC_Os01g63420 | *CORONATINE INSENSITIVE PROTEIN1* | 1.05 | 0.010 |
| LOC_Os08g33160 | *JASMONATE ZIM-DOMAIN CONTAINING PROTEIN* | 1.70 | 0.017 |
| LOC_Os04g55920 | *JASMONATE ZIM-DOMAIN CONTAINING PROTEIN* | 2.08 | 0.008 |
| LOC_Os10g25290 | *JASMONATE ZIM-DOMAIN CONTAINING PROTEIN* | 1.55 | 0.009 |
| LOC_Os09g26780 | *JASMONATE ZIM-DOMAIN CONTAINING PROTEIN* | 1.08 | 0.005 |
| LOC_Os04g32480 | *JASMONATE ZIM-DOMAIN CONTAINING PROTEIN* | 2.51 | 0.012 |
| LOC_Os03g08320 | *JASMONATE ZIM-DOMAIN CONTAINING PROTEIN* | 1.39 | 0.009 |
| LOC_Os03g28940 | *JASMONATE ZIM-DOMAIN CONTAINING PROTEIN* | 1.83 | 0.017 |
| LOC_Os07g35870 | *TRANSCRIPTION FACTOR MYC2* | 1.14 | 0.014 |
| LOC_Os10g42430 | *TRANSCRIPTION FACTOR MYC2* | 1.05 | 0.013 |
| LOC_Os02g02480 | *TRANSCRIPTION FACTOR MYC2* | 1.29 | 0.008 |

**Table S3.** Primers used in this study

| **Primer** | **Length** | **Sequence** | **Location corresponding to nucleotide positions** | **Orientation** |
| --- | --- | --- | --- | --- |
| ML1062 | 20-mer | 5'-AAGGCCGCTCGGGAGCTTGT-3' | 322-341 in *OsACBP5* mRNA (GenBank accession number NM_001056063) | Forward |
| ML1063 | 24-mer | 5'-CTATCAAGCTTTGACAGGGCAGCC-3' | 1305-1328 in *OsACBP5* mRNA (GenBank accession number NM_001056063) | Reverse |
| ML1115 | 20-mer | 5**'**-AGGCCGTCCTCTCTCTGTAT-3**'** | 522–541 in rice *ACTIN* mRNA (GenBank accession number AK100267) | Forward |
| ML1116 | 20-mer | 5**'**-GGGGAGAGCATATCCTTCAT-3**'** | 609–628 in rice *ACTIN* mRNA (GenBank accession number AK100267) | Reverse |
| ML2471 | 29-mer | 5**'**-TAGAAGCTTTGACTTCCTCACGCTCTGCT-3**'** | -1931 – -1905 in 2.2-kb *OsACBP5* 5'-flanking sequence (Locus ID Os03g14000). *Hind*III site underlined. | Forward |
| ML2472 | 29-mer | 5**'**-TAGGGATCCGCTGCACTCCTCTCTCTCTC-3**'** | 280 – 306 in 2.2-kb *OsACBP5* 5'-flanking sequence (Locus ID Os03g14000). *BamH*I site underlined. | Reverse |
| ML2536 | 28-mer | 5**'**-TAGAAGCTTGTACAAAACACACCGTTTA-3’ | -1281 – -1255 in 2.2-kb *OsACBP5* 5'-flanking sequence (Locus ID Os03g14000). *Hind*III site underlined. | Forward |
| ML2538 | 28-mer | 5**'**-TAGAAGCTTCGAAAAACCCCCTGCCTGC-3’ | -46 – -27 in 2.2-kb *OsACBP5* 5'-flanking sequence (Locus ID Os03g14000). *Hind*III site underlined. | Forward |
| ML2572 | 19-mer | 5**'**-CACATGGGTGTCATGGCGG-3’ | -1721 – -1703 in 2.2-kb *OsACBP5* 5'-flanking sequence (Locus ID Os03g14000) | Forward |
| ML2573 | 19-mer | 5**'**-CCGCCATGACACCCATGTG-3’ | -1721 – -1703 in 2.2-kb *OsACBP5* 5'-flanking sequence (Locus ID Os03g14000) | Reverse |
| ML2586 | 20-mer | 5**'**-CACCAACCTGACCCAGAGGC-3’ | -164 – -145 in 2.2-kb *OsACBP5* 5'-flanking sequence (Locus ID Os03g14000) | Forward |
| ML2587 | 20-mer | 5**'**-GCCTCTGGGTCAGGTTGGTG-3’ | -164 – -145 in 2.2-kb *OsACBP5* 5'-flanking sequence (Locus ID Os03g14000) | Reverse |
| ML2812 | 22-mer | 5**'**-TGTACACGGGCAAGCTCCGGCC-3’ | 545 – 566 in *OsNPR1* mRNA (GenBank accession number HM991166) | Forward |
| ML2813 | 22-mer | 5'-GATCTTGAACGCCCACGCCGCG-3’ | 657 – 678 in *OsNPR1* mRNA (GenBank accession number HM991166) | Reverse |
| ML2816 | 22-mer | 5'-GGTGAAGAAGGACTACGACCGC-3’ | 936 – 957 in *OsAOS1* mRNA (GenBank accession number AB116527) | Forward |
| ML2817 | 22-mer | 5'-CCGAACGAGTTGAAGCAGAGC-3’ | 1056 – 1076 in *OsAOS1* mRNA (GenBank accession number AB116527) | Reverse |
| ML3196 | 20-mer | 5**'**-CGCGGGGAACAAGGATAACG-3**'** | 235-254 in *Os07g22710* mRNA (Accession number IPR020642) | Forward |
| ML3197 | 15-mer | 5**'**-GGCGGCGAAGGTGTA-3**'** | 365-379 in *Os07g22710* mRNA (Accession number IPR020642) | Reverse |
| ML3198 | 21-mer | 5**'**-CCTTTCTCCATCACTTCAGCA-3**'** | 7437-7457 in *Os08g35210* mRNA (Accession number IPR013623) | Forward |
| ML3199 | 21-mer | 5**'**-ATGAAGCTCTTCTCGGAACGC-3**'** | 7552-7572 in *Os08g35210* mRNA (Accession number IPR013623) | Reverse |
| ML3200 | 20-mer | 5**'**-ACACGTCGCAGCAGCTGGCG-3**'** | 529-548 in *Os12g28270* mRNA (Accession number IPR006680) | Forward |
| ML3201 | 20-mer | 5**'**-CTCGATGCCGCAGAGGAGGG-3**'** | 646-665 in *Os12g28270* mRNA (Accession number IPR006680) | Reverse |
| ML3202 | 21-mer | 5**'**-GGATCTTGACAAGAAGGTGGC-3**'** | 242-262 in *Os05g05460* mRNA (Accession number IPR011992) | Forward |
| ML3203 | 21-mer | 5**'**-ACATCCCTGATGTTCCTGAGG-3**'** | 353-373 in *Os05g05460* mRNA (Accession number IPR011992) | Reverse |
| ML3204 | 16-mer | 5**'**-GTCTCCACGCTGGTGG-3**'** | 235-250 in *Os06g38970* mRNA (Accession number IPR001611) | Forward |
| ML3205 | 15-mer | 5**'**-TGAGCGACGGCGTTG-3**'** | 353-373 in *Os06g38970* mRNA (Accession number IPR001611) | Reverse |
| ML3206 | 15-mer | 5**'**-TGCGGCTACTTCGGC-3**'** | 341-355 in *Os03g58110* mRNA (Accession number IPR001611) | Forward |
| ML3207 | 15-mer | 5**'**-AGGTCGGTGAGGAGG-3**'** | 457 471 in *Os03g58110* mRNA (Accession number IPR001611) | Reverse |
| ML3208 | 20-mer | 5**'**-ATTCTCGAGTTCTACATGGG-3**'** | 945-964 in *Os02g54600* mRNA (Accession number IPR011009) | Forward |
| ML3209 | 14-mer | 5**'**-CGGCGAGGCGTTGG-3**'** | 1057-1070 in *Os02g54600* mRNA (Accession number IPR011009) | Reverse |
| ML3210 | 23-mer | 5**'**-AGAACAGCGACGGCTCCGGCAAG-3**'** | 1403-1425 in *Os02g08440* mRNA (Accession number IPR003657) | Forward |
| ML3211 | 22-mer | 5**'**-GATCGATCGAACTCCGCCATGG-3**'** | 1801-1822 in *Os02g08440* mRNA (Accession number IPR003657) | Reverse |
| ML3212 | 20-mer | 5**'**-CGAGTCCGATCACTAGCCTC-3**'** | 1145-1164 in *Os03g53050* mRNA (Accession number IPR003657) | Forward |
| ML3213 | 16-mer | 5**'**-GAAGGCAATGCCGGGT-3**'** | 1349-1364 in *Os03g53050* mRNA (Accession number IPR003657) | Reverse |
| ML3214 | 15-mer | 5**'**-GTGCGGCCACTACAC-3**'** | 443-457 in *Os07g03600* mRNA (Accession number IPR014044) | Forward |
| ML3215 | 20-mer | 5**'**-CACAATCCGGCTACATAGAT-3**'** | 652-671 in *Os07g03600* mRNA (Accession number IPR014044) | Reverse |
| ML3216 | 22-mer | 5**'**-GCCTACATTGAATCTGAACTTC-3**'** | 4141-4162 in *Os01g20900* mRNA (Accession number IPR000719) | Forward |
| ML3217 | 20-mer | 5**'**-CTGCTGAGGGTTTTATGTGG-3**'** | 4318-4337 in *Os01g20900* mRNA (Accession number IPR000719) | Reverse |
| ML3218 | 20-mer | 5**'**-GCAAGGGTAAATCTCCAGGG-3**'** | 1430-1449 in *Os03g63140* mRNA (Accession number IPR008700) | Forward |
| ML3219 | 21-mer | 5**'**-GGTGACTCAGCATATGGTTTT-3**'** | 1605-1625 in *Os03g63140* mRNA (Accession number IPR008700) | Reverse |
| ML3220 | 16-mer | 5**'**-AGCCTCTTCCACGGCA-3**'** | 978-993 in *Os03g20550* mRNA (Accession number IPR003657) | Forward |
| ML3221 | 20-mer | 5**'**-CCTGCATCTACTGTCTCTTC-3**'** | 1106-1125 in *Os03g20550* mRNA (Accession number IPR003657) | Reverse |
| ML3222 | 16-mer | 5**'**-GCTGATGCCGCATGAA-3**'** | 2416-2431 in *Os02g33180* mRNA (Accession number IPR007051) | Forward |
| ML3223 | 17-mer | 5**'**-TGAAACACCACGCACAC-3**'** | 2601-2617 in *Os02g33180* mRNA (Accession number IPR007051) | Reverse |
| ML3224 | 17-mer | 5**'**-GGTCTCCGGTGACGATC-3**'** | 1402-1418 in *Os08g38990* mRNA (Accession number IPR003657) | Forward |
| ML3225 | 22-mer | 5**'**-CCTCCATATAAACCACATGAGT-3**'** | 1535-1556 in *Os08g38990* mRNA (Accession number IPR003657) | Reverse |
| ML3226 | 15-mer | 5**'**-CCCTCCACTCCGGCC-3**'** | 236-250 in *Os01g56200* mRNA (Accession number IPR021094) | Forward |
| ML3227 | 16-mer | 5**'**-CCCCTCTGCTCGTCTC-3**'** | 394-409 in *Os01g56200* mRNA (Accession number IPR021094) | Reverse |
| ML3228 | 18-mer | 5**'**-CGAAGTAACCAAGGCTGT-3**'** | 1932-1949 in *Os04g54474* mRNA (Accession number IPR004827) | Forward |
| ML3229 | 19-mer | 5**'**-CCTCATGCTTAGGATGCAC-3**'** | 2129-2147 in *Os04g54474* mRNA (Accession number IPR004827) | Reverse |
| ML3230 | 20-mer | 5**'**-GGCTGCTGAATACTCTGAGA-3**'** | 744-763 in *Os05g50890* mRNA (Accession number IPR004993) | Forward |
| ML3231 | 13-mer | 5**'**-TGGGAGGGGGGCG-3**'** | 925-937 in *Os05g50890* mRNA (Accession number IPR004993) | Reverse |
| ML3232 | 20-mer | 5**'**-GGTGTGTGTGTTGTGTGTGT-3**'** | 1303-1322 in *Os03g15880* mRNA (Accession number GO:0006950) | Forward |
| ML3233 | 21-mer | 5**'**-ACTGAGTTAAGCTCAAATTGC-3**'** | 1517-1537 in *Os03g15880* mRNA (Accession number GO:0006950) | Reverse |
| ML3234 | 20-mer | 5**'**-CCCTTGGATGCCCATCTTAT-3**'** | 2234-2253 in *Os04g55920* mRNA (Accession number IPR010399) | Forward |
| ML3235 | 21-mer | 5**'**-CTGGCGGCACTGAGTCATAAA-3**'** | 2454-2474 in *Os04g55920* mRNA (Accession number IPR010399) | Reverse |
| ML3236 | 22-mer | 5**'**-GTACTGGAACAAAACCACTGGC-3**'** | 3048-3069 in *Os02g02480* mRNA (Accession number IPR011598) | Forward |
| ML3237 | 17-mer | 5**'**-CACCAGATGCCGTGGAC-3**'** | 3195-3211 in *Os02g02480* mRNA (Accession number IPR011598) | Reverse |
